# Sulfonylurea Receptor 1, Transient Receptor Potential Cation Channel Subfamily M Member 4, and KIR6.2:Role in Hemorrhagic Progression of Contusion

**DOI:** 10.1089/neu.2018.5986

**Published:** 2019-03-15

**Authors:** Volodymyr Gerzanich, Jesse A. Stokum, Svetlana Ivanova, Seung Kyoon Woo, Orest Tsymbalyuk, Amit Sharma, Fatih Akkentli, Ziyan Imran, Bizhan Aarabi, Juan Sahuquillo, J. Marc Simard

**Affiliations:** ^1^ Department of Neurosurgery, University of Maryland School of Medicine, Baltimore, Maryland.; ^2^ Neurotraumatology and Neurosurgery Research Unit, Vall d'Hebron University Hospital, Universitat Autònoma de Barcelona, Barcelona, Spain.; ^3^ Department of Neurosurgery, Vall d'Hebron University Hospital, Universitat Autònoma de Barcelona, Barcelona, Spain.; ^4^ Department of Pathology, University of Maryland School of Medicine, Baltimore, Maryland.; ^5^ Department of Physiology, University of Maryland School of Medicine, Baltimore, Maryland.

**Keywords:** brain contusion, brain swelling, glibenclamide, hemorrhagic progression of contusion, K_ATP_, SUR1-TRPM4, traumatic brain injury

## Abstract

In severe traumatic brain injury (TBI), contusions often are worsened by contusion expansion or hemorrhagic progression of contusion (HPC), which may double the original contusion volume and worsen outcome. In humans and rodents with contusion-TBI, sulfonylurea receptor 1 (SUR1) is upregulated in microvessels and astrocytes, and in rodent models, blockade of SUR1 with glibenclamide reduces HPC. SUR1 does not function by itself, but must co-assemble with either KIR6.2 or transient receptor potential cation channel subfamily M member 4 (TRPM4) to form K_ATP_ (SUR1-KIR6.2) or SUR1-TRPM4 channels, with the two having opposite effects on membrane potential. Both KIR6.2 and TRPM4 are reportedly upregulated in TBI, especially in astrocytes, but the identity and function of SUR1-regulated channels post-TBI is unknown. Here, we analyzed human and rat brain tissues after contusion-TBI to characterize SUR1, TRPM4, and KIR6.2 expression, and in the rat model, to examine the effects on HPC of inhibiting expression of the three subunits using intravenous antisense oligodeoxynucleotides (AS-ODN). Glial fibrillary acidic protein (GFAP) immunoreactivity was used to operationally define core versus penumbral tissues. In humans and rats, GFAP-negative core tissues contained microvessels that expressed SUR1 and TRPM4, whereas GFAP-positive penumbral tissues contained astrocytes that expressed all three subunits. Förster resonance energy transfer imaging demonstrated SUR1-TRPM4 heteromers in endothelium, and SUR1-TRPM4 and SUR1-KIR6.2 heteromers in astrocytes. In rats, glibenclamide as well as AS-ODN targeting SUR1 and TRPM4, but not KIR6.2, reduced HPC at 24 h post-TBI. Our findings demonstrate upregulation of SUR1-TRPM4 and K_ATP_ after contusion-TBI, identify SUR1-TRPM4 as the primary molecular mechanism that accounts for HPC, and indicate that SUR1-TRPM4 is a crucial target of glibenclamide.

## Introduction

The most common mass lesions associated with traumatic brain injury (TBI) are hemorrhagic brain contusions (BCs), also called traumatic intracerebral hematomas, which occur in 13–35% of patients following moderate-to-severe TBI.^1–3^ BCs remain a serious clinical problem because of their associated morbidity. Mechanical loading (i.e., applied mechanical forces) physically destroy brain tissues and trigger mechanosensitive processes that initiate secondary injury cascades in the surrounding penumbra, most importantly, contusion expansion, or “hemorrhagic progression of contusion” (HPC). HPC is not simply an intracerebral hematoma that expands from a point source, compressing normal brain, but instead is characterized by a progressive increase in the volume of brain undergoing hemorrhagic transformation.^4,5^

HPC occurs during the first few hours after trauma, and the pericontusional zone or “penumbra” is a key driver of this progression. An expanding hemorrhage post-TBI may be linked to coagulopathy or platelet dysfunction,^6^ but absent these causes, HPC is attributed to the catastrophic structural failure of microvessels.^4,5^ Dysfunction or death of microvascular endothelial cells in the penumbra results in the fragmentation of microvessels, leading to the formation of petechial hemorrhages.^7^ Experimental work has shown that, as microvascular fragmentation progresses outward from the initial site of contusion, petechial hemorrhages coalesce, eventually doubling the original contusion volume over the course of 12 h after trauma.^4^ In humans with BCs, HPC occurs in 50–75% of cases, hemorrhagic lesion volumes expand 50% or more with an average expansion rate of 0.7 mL per hour, and increased expansion rates are significantly associated with poor outcome or death.^3,8,9^

HPC is of vital clinical importance. HPC not only destroys brain tissue, but it also causes brain swelling, the strongest prognostic indicator of poor outcome in TBI.^10,11^ In TBI, brain swelling arises from the combined space-occupying effects of extracellular edema fluid, cellular swelling, extravasated blood, and other processes. Edema, which is recognized as a major contributor to brain swelling, can be treated using osmotherapeutic agents such as mannitol and hypertonic saline. By contrast, extravasated blood, which arises from both the initial contusion and from HPC, and which also is a major contributor to brain swelling, currently has no clinical treatment. Reducing HPC could significantly impact brain swelling in BC.

The ion channel regulatory subunit, sulfonylurea receptor 1 (SUR1), is strongly linked to TBI, especially BC. SUR1 protein and *Abcc8* messenger RNA (mRNA), which encodes SUR1, are transcriptionally upregulated at the site of injury in animal models of TBI and in humans with TBI.^4,12,13^ SUR1 upregulation is found in microvascular endothelium, astrocytes, and neurons. SUR1 is held to be responsible for microvascular failure leading to edema and HPC, since edema, capillary fragmentation and HPC are reduced by blocking SUR1 with glibenclamide.^4,14–17^ In a recent study in humans, Jha and colleagues showed that the level of SUR1 in the ventricular cerebrospinal fluid was a good biomarker of brain swelling identified on the computed tomography (CT) scan.^18^ Also, the same group showed that ABCC8 genetic variability in patients with TBI and regionally clustered single nucleotide polymorphisms are associated with post-traumatic brain swelling.^19,20^ Recently, pharmacological inhibition of SUR1 was shown in human clinical trials to reduce brain swelling after ischemic stroke^21^ and to reduce HPC after TBI.^22^

The unique molecular biology of SUR1 has hindered a full understanding of its pathological role in TBI. SUR1 is a member of the adenosine triphosphate (ATP) binding cassette (ABC) transporter superfamily. SUR1 does not function by itself but instead modulates the properties of distinct pore-forming ion channels by participating in heterologous co-associations. In different cell types, SUR1 co-assembles either with the inwardly rectifying K^+^ (KIR) 6.2 channel to form K_ATP_ channels,^23–25^ or with transient receptor potential melastatin 4 (TRPM4) to form SUR1-TRPM4 channels.^26^ Notably, these two channels have opposite physiological effects—upon activation, K_ATP_ channels mediate K^+^ efflux and cell hyperpolarization, whereas SUR1-TRPM4 channels mediate Na^+^ influx and cell depolarization. In TBI, little is known about SUR1's potential pore-forming subunit partners. Two recent reports have shed important light on this question, but these reports yielded unexpected findings: Gorse and colleagues^27^ reported that TRPM4 was upregulated in a rodent model of TBI, especially in astrocytes, whereas Castro and colleagues^28^ reported that KIR6.2 was upregulated in humans with TBI, again especially in astrocytes. Given the opposite functional effects of the two channels, we thought it important to revisit this question of astrocyte expression of SUR1-regulated channels post-TBI. Also, given the critical role of SUR1 in HPC post-TBI, we thought it important to determine which of the SUR1-regulated channels was linked to HPC.

Here, we studied the expression and assembly of SUR1, TRPM4 and KIR6.2 in brain tissues from humans with TBI and from a rat model of TBI, with a particular focus on astrocyte expression of these channels. To address the function of the three subunits in HPC, we studied the effects of antisense-mediated gene knockdown of the three subunits in a rat model of contusion-TBI. Here, we report that, indeed, both TRPM4 and KIR6.2 are upregulated post-TBI in astrocytes of both species, where they co-assemble with SUR1 to form heteromers of SUR1-TRPM4 and SUR1-KIR6.2. Also, we report that inhibiting the expression of SUR1 and TRPM4, but not KIR6.2, reduces HPC post-TBI.

## Methods

### Human tissues

All cases were patients at the R. Adams Cowley Shock Trauma Center, Baltimore; the cases reported here are distinct from the cohort from Barcelona reported earlier.^13,28^ The tissue collection protocol was approved by the Institutional Review Board (IRB) of the University of Maryland School of Medicine. Under this IRB protocol, brain tissue biopsy specimens were obtained from 16 patients with non-ballistic, closed head injury, contusion-TBI who underwent decompressive craniectomy (by BA or JMS) for elevated intracranial pressure^29^ between January 12, 2013, and August 3, 2014. All had CT scans exhibiting hemorrhagic contusions with mass effect. Patients were (mean ± standard deviation) 48 ± 20 years of age, and 81% were male. The mechanism of injury was motor vehicle accident, pedestrian struck, or assault. Time to surgery was 58 ± 85 h (range, 2–280) after TBI.

In each case, one or two biopsy specimens, 0.5–1 cm^3^, were obtained from what appeared grossly to be non-necrotic brain at the interface between contused brain and normal-appearing brain. Following biopsy, specimens were placed immediately in 4% formaldehyde for immersion fixation. Tissues were cryoprotected with 30% sucrose, embedded in optimum cutting temperature (OCT) compound, frozen on dry ice, and cryosectioned (10 μm).

For control tissues, five postmortem specimens were obtained from three men and two women who died rapidly from non-neurological diseases, including pneumonia, status asthmaticus, and rupture of aortic aneurysm.^30^ The postmortem interval ranged from 18–74 h. The average age of control subjects was 51.3 ± 7.0 years.

### Antibody validation

The custom anti-SUR1 and anti-TRPM4 antibodies used for immunolabeling and immunoFRET in this study were validated previously using lysates from appropriate SUR1 and TRPM4 expression systems, tissues from wild-type, *Trpm4–/–* and *Abcc8–/–* mice, and using mass spectrometry of immunoisolated proteins,^26^ in accordance with recommendations for antibody validation by Uhlen and colleagues.^31^ Here, we further validated the specificity of the custom anti-SUR1 antibody for immunohistochemistry of human tissues by comparing immunolabeling of adjacent sections using our custom polyclonal antibody, a monoclonal antibody (S289-16; Novus Biologicals, Littleton, CO) and a commercial polyclonal antibody (sc-5789; Santa Cruz Biotechnology, Dallas, TX).

We evaluated the suitability of three commercial anti-KIR6.2 antibodies using myc-tagged KIR6.x (myc-KIR6.2 or myc-KIR6.1) plasmids expressed in COS-7 cells. Myc-KIR6.2 and myc-KIR6.1 plasmids were constructed as follows. Complementary DNA clones encoding mouse *Kcnj11* (KIR6.2) and *Kcnj8* (KIR6.1) were obtained from Dr. Shyng (Oregon Health and Science University, Portland, OR) and DNASU Plasmid Repository (Tempe, AZ), respectively, and used as templates for polymerase chain reaction (PCR). The amplified PCR products were cloned into pCMV-Tag3C vector (Agilent Technologies, Santa Clara, CA) for expression of myc-KIR6.x. The myc-KIR6.x plasmids were verified by DNA sequencing prior to transfection.

Whole–cell lysates and myc-KIR6.2 immunoprecipitates were immunoblotted using anti-myc antibody (sc-40; Santa Cruz Biotechnology) or one of three anti-KIR6.2 antibodies: 1) GTX80493, an anti-human (N-terminus of KIR6.2), rabbit polyclonal antibody from GeneTex (Irvine, CA); 2) sc-11226, an anti-human (N-terminus of KIR6.2) goat polyclonal antibody from Santa Cruz Biotechnology; or 3) sc-11228, an anti-human (internal region of KIR6.2) goat polyclonal antibody from Santa Cruz Biotechnology.

**FIG. 1.**
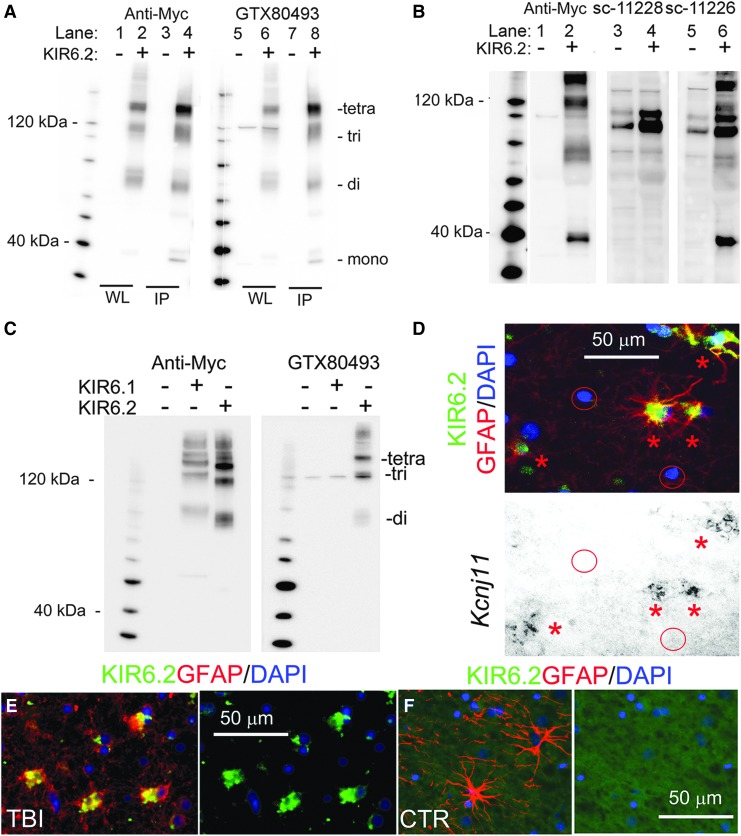
KIR6.2 antibody validation. (**A)** Whole–cell lysate (WL) or anti-myc immunoprecipitate (IP) were prepared from COS-7 cells transfected with myc-KIR6.2 or empty vector; immunoblot with anti-myc antibody showed no bands with expression of empty vector (lanes 1, 3), and showed KIR6.2 monomers and multimers with KIR6.2 expression (lanes 2, 4); immunoblot with GTX80493 anti-KIR6.2 antibody showed a single faint nonspecific band in WL immunoblot of empty vector (lane 5), and showed KIR6.2 monomers and multimers with KIR6.2 expression (lanes 6, 8); representative of three experiments. (**B)** Whole lysate was prepared from COS-7 cells transfected with myc-KIR6.2 or empty vector; immunoblot with anti-myc antibody showed no bands with expression of empty vector (lane 1), and showed KIR6.2 monomers and multimers with KIR6.2 expression (lane 2); immunoblot with sc-11228 anti-KIR6.2 antibody showed multiple nonspecific bands with expression of empty vector (lane 3), and no KIR6.2 with expression of KIR6.2 (lane 4); immunoblot with sc-11226 anti-KIR6.2 antibody showed multiple nonspecific bands with expression of empty vector (lane 5), and showed KIR6.2 multimers with expression of KIR6.2 (lane 6); representative of three experiments. **(C)** Whole lysate was prepared from COS-7 cells transfected with empty vector, or myc-KIR6.1 or myc-KIR6.2; immunoblot with anti-myc antibody showed no bands with expression of empty vector, and showed KIR6.1 or KIR6.2 multimers with KIR6.1 or KIR6.2 expression, as indicated; immunoblot with GTX80493 anti-KIR6.2 antibody showed a single faint nonspecific band with empty vector and with KIR6.1 expression, and showed multimers with KIR6.2 expression, as indicated; representative of three experiments. **(D)** Combined *in situ* hybridization and double immunolabeling of the same tissue section from human brain after TBI. Upper panel: superimposed double immunolabeling for KIR6.2 (GTX80493, GeneTex; green) and glial fibrillary acidic protein (GFAP; red) showed astrocytes that express KIR6.2 (asterisks); in the same field, other cells identified by 4′,6-diamidino-2-phenylindole staining of nuclei showed no immunoreactivity for GFAP or KIR6.2 (red circles). Lower panel: *in situ* hybridization for *Kcnj11* in the same tissue section showed positive signal within the same astrocytes that express KIR6.2 protein (asterisks), and no signal in cells that express no KIR6.2 protein (circles); representative of five experiments. **(E)** Double immunolabeling for KIR6.2 using APC-020 (Alomone Labs; green) and GFAP (red) in tissues from the same case as in (D) confirmed the specificity of the anti-KIR6.2 antibody, GTX80493. **(F)** Double immunolabeling for KIR6.2 (GTX80493, GeneTex; green; green) and GFAP (red) in control (uninjured) cortical tissue showed that GTX80493 exhibited no immunoreactivity when used to label control GFAP+ tissues.

Anti-myc immunoblot of whole–cell lysates and of myc-KIR6.2 immunoprecipitate from cells transfected with empty vector yielded no bands (Fig. 1A; lanes 1, 3). Anti-myc immunoblots of whole–cell lysates and myc-KIR6.2 immunoprecipitate from cells transfected with myc-KIR6.2 showed bands at ∼75 kDa, ∼110 kDa, and ∼150 kDa (Fig. 1A; lanes 2, 4). An additional band at ∼38 kDa was observed in the anti-myc immunoblot of myc-KIR6.2 immunoprecipiate (Fig. 1A; lane 4). These four bands correspond to the monomer and multimers of KIR6.2.^32^

The antibody from GeneTex yielded an immunoblot that was nearly identical to that obtained with anti-myc antibody (Fig. 1A; lanes 5–8). GTX80493 detected all multimers of KIR6.2 (Fig. 1A; lane 8). GTX80493 showed one faint nonspecific band ∼120 kDa in the absence of KIR6.2 expression (Fig. 1A; lane 5).

The antibodies from Santa Cruz Biotechnology were evaluated using only whole–cell lysates (Fig. 1B; lanes 1, 2). When used to immunoblot lysates that did not contain KIR6.2, the sc-11228 immunoblot showed many nonspecific bands (Fig. 1B; lane 3), and detected KIR6.2 only very poorly (Fig. 1B; lane 4). Similarly, sc-11226 showed many nonspecific bands (Fig. 1B; lane 5), although it did detect all KIR6.2 multimers (Fig. 1B; lane 6).

We also evaluated the ability of GTX80493 to distinguish between KIR6.2 and KIR6.1 (both of which can be expressed by astrocytes; see Discussion). COS7 cells were used to express myc-KIR6.2 or myc-KIR6.1, as above. Immunoblot using anti-myc antibody confirmed the expression of both (Fig. 1C). Immunoblot using GTX80493 again confirmed detection of KIR6.2 multimers, but KIR6.1 was not detected. As noted above, GTX80493 showed one faint nonspecific band ∼120 kDa in both the absence and presence of KIR6.x expression (Fig. 1C).

To further validate GTX80493 specifically for immunohistochemistry, we used it to co-localized expression of KIR6.2 protein and *Kcnj11* mRNA in brain tissue sections from contusion-TBI patients. *In situ* hybridization was performed for *Kcnj11* mRNA, and the same tissue section was double-immunolabeled for glial fibrillary acidic protein (GFAP) and for KIR6.2 using GTX80493 (see Methods). Immunolabeling showed GFAP-expressing stellate cells with prominent expression of KIR6.2, with the same cells also exhibiting *Kcnj11* mRNA (Fig. 1D; asterisks). In the same field, other cells were present, as identified by nuclear staining with 4′,6-diamidino-2-phenylindole (DAPI); these cells showed no immunoreactivity with GTX80493, and no *Kcnj11* mRNA (Fig. 1D; circles). Using a different anti-KIR6.2 antibody (APC-020; Alomone Labs) that was validated previously^28^ confirmed astrocyte-specific expression of KIR6.2 in the same tissue specimen (Fig. 1E). GTX80493 exhibited no immunoreactivity when used to label control (uninjured) GFAP+ tissues (Fig. 1F).

All immunohistochemistry for KIR6.2 reported in this manuscript was conducted using GTX80493.

### Histology, immunolabeling, and immunoFRET of human tissues

Human sections were stained with hematoxylin and eosin using routine techniques, and were examined with light microscopy. For terminal deoxynucleotidyl transferase dUTP nick end labeling (TUNEL) assay, cryosections were processed according to kit instructions (C10617; Thermo Fisher Scientific Inc.).

For fluorescence immunohistochemistry, cryosections were blocked 1 h in 2% donkey serum (D9663; Sigma-Aldrich, St. Louis, MO) and 0.2% Triton X-100. After rinsing, sections were incubated overnight at 4°C with a primary antibody: goat ant-SUR1 (1:500; custom antibody,^26^ mouse monoclonal anti-SUR1 (1:100; S289-16; Novus Biologicals), goat anti-SUR1 (C-16; 1:200; sc-5789; Santa Cruz Biotechnology, Santa Cruz, CA), chicken anti-TRPM4 (1:500; custom antibody), goat anti-TRPM4 (G-20; 1:200; sc-27540; Santa Cruz Biotechnology), or KIR6.2 (1:100; GTX80493; GeneTex, Irvine, CA). After labeling for one of the channel subunits, double immunolabeling was performed using either: CY3-conjugated, monoclonal anti-GFAP (1:500; C9205; Sigma-Aldrich), or rabbit anti-collagen IV (1:200; ab6586; Abcam, Cambridge, MA), or mouse anti-CD68 (ED1; 1:100; MAB1435; Millipore Sigma, Burlington, MA), to analyze cell-specific expression. For visualization, we used fluorescent-labeled species-appropriate secondary antibodies (1:500, Alexa Fluor 488 [green] and Alexa Fluor 555 [red]; Invitrogen/Molecular Probes, Eugene, OR) at room temperature. Sections were cover-slipped using ProLong Gold antifade reagent containing the nuclear stain, DAPI (Invitrogen). Immunolabeled sections were visualized using epifluorescence microscopy (Nikon Eclipse 90i; Nikon Instruments Inc., Melville, NY). Specific labeling was defined as fluorescence intensity twice that of background. Controls included incubation with pre-immune serum, when available, omission of primary antibodies, and testing cell-specific antibodies (anti-GFAP, anti-CD68) to assure no cross-reactivity with SUR1, TRPM4, or KIR6.2.

To demonstrate formation of SUR1-TRPM4 and SUR1-KIR6.2 heteromers, we performed antibody-based Förster Resonance Energy Transfer (immunoFRET),^33^ using methods we described.^26,34,35^ To study SUR1-TRPM4 heteromers, we used rabbit anti-SUR1 (1:200; custom antibody) and goat anti-TRPM4 (G-20; 1:200; sc-27540; Santa Cruz Biotechnology) primary antibodies, along with donkey anti-rabbit CY5 (1:100) and mouse anti-goat CY3 (1:100) (the Jackson Laboratory) secondary antibodies. To study SUR1-KIR6.2 heteromers, we used goat anti-SUR1 (1:400; custom antibody) and rabbit anti-KIR6.2 (1:50: GTX80493; Genetex) primary antibodies, along with donkey anti-rabbit CY5 (1:100) and mouse anti-goat CY3 (1:100; the Jackson Laboratory) secondary antibodies. Sections were first immunolabeled using a mixture of the two primary antibodies, with incubation performed overnight at 4°C. After washing with phosphate-buffered saline (PBS), the sections were incubated with a mixture of the two secondary antibodies for 1 h, rinsed and cover-slipped. FRET fluorescence imaging was analyzed on a commercial laser scanning microscope combination system, LSM510 Meta (Zeiss, Jena, Germany) with an Axiovert inverted microscope and a 63 × oil immersion lens. The following settings were used throughout the experiments: for Cy3, excitation, 543 nm; detection, 565–615 nm; for Cy5, excitation, 633 nm; detection, 650–704 nm. The images were captured and processed with the LSM Image Examiner software (Zeiss), with an integration time of 5 msec/pixel. ROIs were three rectangles, 225 × 180 μm, positioned within GFAP+ tissues. Controls studied here included omission of primary antibodies and study of uninjured control brain tissues; additional controls for immunoFRET validation used a COS-7 cell expression system, as described.^26^

### Immunohistochemistry analysis

Channel subunit expression was assessed in all cases, but was quantified only in cases with GFAP-positive specimens. Unbiased measurements of signal intensity within regions of interest (ROIs) were obtained using NIS-Elements AR software (Nikon Instruments, Melville, NY). Images were collected from sections double immunolabeled for GFAP and SUR1 or TRPM4 or KIR6.2. The GFAP channel was used to define ROIs (pixel intensity >2 × background). The GFAP-defined ROIs were used to quantify astrocyte-specific expression of SUR1, TRPM4, or KIR6.2, with the % ROI calculated by dividing the number of positive pixels (> 2 × background) for individual channel subunits by the total number of GFAP+ pixels in the ROI. For each case, three representative areas (410 × 325 μm ROI) were analyzed and averaged.

### In situ hybridization

For *in situ* hybridization, cryosections were processed with digoxigenin-labeled probes against *Abcc8* (antisense: 5′-TGCAGGGGTCAGGGTCAGGGCGCTGTCGGTCCACTTGGCCAGCCAGTA-3′), TRPM4 (antisense: 5′-CCGAGAGTGGAATTCCCGGATGAGGCGGTAACGCTGC-3′), or KIR6.2 (antisense: 5′-CCATGCGCCCCCCAAAGCCAATAGTCACTTGGACCTCAAT-3′), supplied by IDT (Skokie, IL). *In situ* hybridization was performed on 10-μm sections and controls using an IsHyb In Situ Hybridization (ISH) Kit (Biochain Institute, Inc., Newark, CA) according to the manufacturer's protocol. Sections were incubated in diethyl pyrocarbonate (DEPC)–treated PBS and fixed in 4% paraformaldehyde in PBS for 20 min. After being rinsed twice with DEPC-PBS, the slides were treated with 10 μg/mL proteinase K at 37°C for 10 min. Slides were then washed in DEPC-PBS, rinsed with DEPC-H_2_O, and prehybridized with ready-to-use prehybridization solution (BioChain Institute, Newark, CA) for 3 h at 50°C. The DIG-labeled probe was diluted in hybridization buffer (BioChain Institute, Newark, CA) and applied at 4 ng/μL. Sections then were incubated at 45°C for 16 h. Post-hybridization washing and immunological detection, using anti-DIG-alkaline phosphatase (AP) with NBT/BCIP as substrates were performed as recommended by the manufacturer. AP-conjugated anti-DIG antibodies (1:100 PBS diluted, BioChain Institute, Newark, CA), were incubated with slides for 2 h. Finally, slides were rinsed in distilled H_2_O and then were immunolabeled for the corresponding protein using a fluorescent secondary antibody, as above.

### Rat experiments

#### Ethics statement

We certify that all applicable institutional and governmental regulations concerning the ethical use of animals were followed during the course of this research. Animal experiments were performed under a protocol approved by the Institutional Animal Care and Use Committee of the University of Maryland School of Medicine, and in accordance with the relevant guidelines and regulations as stipulated in the United States National Institutes of Health Guide for the Care and Use of Laboratory Animals. All efforts were made to minimize the number of animals used and their suffering.

#### Subjects and surgical procedure for contusion-TBI

Male Wistar rats, ages 12–16 weeks (325–400 g; Harlan, Indianapolis, IN), were anesthetized (60 mg/kg ketamine plus 7.5 mg/kg xylazine, intraperitoneally) and allowed to breathe room air spontaneously. Core temperature was maintained at 37°C with an isothermal pad (Deltaphase; Braintree Scientific, Braintree, MA). Oxygen saturation and heart rate were monitored using a pulse oximeter (Mouse Ox™; STARR Life Sciences Corp., Oakmont, PA). Surgical incision sites were prepared using iodine and alcohol, and a sterile environment was maintained throughout the procedure. Rats were mounted in a stereotactic apparatus (Stoelting Co., Wood Dale, IL). Lidocaine solution (2%) was injected prior to making an incision. A midline scalp incision was made to expose the skull. A circular bone incision positioned 2 mm anterior to lambda and 1 mm lateral to midline was made using a high-speed drill with a 1 mm diamond burr (Dremel, model 732), to create a 6-mm diameter craniotomy in the left parietal region. After removing the bone flap, contusion-TBI was induced using a controlled cortical impact device (Impact One impactor system; 39463920; Leica Biosystems, Buffalo Grove, IL) with the following parameter settings: 5 mm diameter impactor, angled 20° from vertical, 4.5 mm tissue displacement from the dural surface, 1 m/sec velocity, 200 msec dwell time. The bone flap was then repositioned and cemented in place using a cyanoacrylate adhesive (Loctite^®^ 454 Prism Instant Adhesive Gel plus Insta-Set accelerator). For analgesia, we used buprenorphine (0.05 mg/kg) 15 min before surgery then every 12 h for 24 h.

#### Series

Four series of rats with contusion-TBI were studied. In series 1, five contusion-TBI rats were euthanized at each of four time-points: 6, 12, 24 and 72 h, with five uninjured rats serving as controls; these rats were used for immunohistochemistry. In series 2, five contusion-TBI rats received no treatment and were euthanized at 15 min to measure the hemorrhagic lesion area (primary hemorrhage before HPC) and hemispheric swelling. In series 3, 18 contusion-TBI rats were administered either glibenclamide (nine rats) or vehicle (nine rats). In series 4, 53 contusion-TBI rats were administered either antisense-oligodeoxynucleotide (AS-ODN) targeting a channel subunit, or scrambled ODN (Scr-ODN) or sense ODN (SE-ODN) as control, including AS-ODN targeting SUR1 (12 rats, with 10 Scr-ODN controls), TRPM4 (seven rats, with eight SE-ODN controls) or KIR6.2 (eight rats, with eight SE-ODN controls). Rats in series 3 and series 4, were euthanized at 24 h to measure the hemorrhagic lesion area (primary hemorrhage plus secondary hemorrhage after HPC) and hemispheric swelling.

#### Treatments

The drug formulation for glibenclamide (#G2539; Sigma) in dimethyl sulfoxide was as described.^36^ Treatment consisted of administering a single loading dose of glibenclamide (10 μg/kg) or an equivalent volume of vehicle intraperitoneally within 10 min of trauma, as well as continuous infusion via mini-osmotic pump (Alzet 2001, 1.0 μL/h; Alzet Corp, Cupertino, CA) beginning at the end of surgery, resulting in delivery of 400 ng/h or an equivalent volume of vehicle subcutaneously until euthanasia. The dose of glibenclamide used here has been shown to not result in hypoglycemia.

Anti-SUR1 (5′-GGCCGAGTGGTTCTCGGT-3′) and anti-TRPM4 (5'-TTGTGGTAACACTCTCCAAA-3′) AS-ODNs were described and validated previously in rats *in vivo*.^37–39^ These AS-ODNs were shown to result in >60% suppression of protein expression as well as highly significant reductions in progressive hemorrhagic necrosis in a contusion spinal cord injury model. Anti-KIR6.2 (5′-CCTTTCGGGACAGCATGGCT-3′) AS-ODN was described and validated previously in rats *in vivo*.^40^ This AS-ODN was shown to result in 80% suppression of *Kcnj11* mRNA expression in the basal ganglia. ODNs, which were phosphorothioated at 4 distal bonds at both the 5′ and 3′ ends to protect against endogenous nucleases,^41^ were obtained commercially (Sigma-Aldrich). Prior to surgery, mini-osmotic pumps (Alzet 2001D, 8 μL/h; Durect Corp.) fitted with jugular vein catheters were loaded with ODN and primed. ODN solutions were prepared in sterile normal saline (NS; 4 mg/mL). Immediately after trauma, the jugular vein was catheterized, a loading dose of ODN (400 μg in 100 μL) was administered intravenously, and the pump catheter was inserted into the vein with the pump implanted subcutaneously in the dorsal thorax, to obtain intravenous delivery of ODN at a rate of 32 μg /h until euthanasia, yielding a total dose delivered of ∼1.2 mg/24 h.

#### Exclusions

In all series, there was no unplanned mortality, and no animal was excluded for any reason.

### Hemorrhagic lesion area and hemispheric swelling

At the designated time after trauma, rats were euthanized by an intraperitoneal injection of pentobarbital (> 100 mg/kg), then underwent transcardial perfusion with NS (60 mL) followed by 10% neutral buffered formalin (50 mL; Sigma-Aldrich). Immediately after perfusion-fixation, brains were harvested and bisected coronally at the contusion epicenter using a pre-chilled (-20°C) rat brain coronal matrix (Ted Pella Inc., Redding, CA). The exposed coronal faces were imaged on a flatbed scanner to obtain high resolution digital images (red-green-blue [RGB]; 600 dpi).

To obtain unbiased measurements of hemorrhagic lesion area, we used an algorithm-based segmentation protocol similar to what we used previously for TTC-stained sections after ischemia,^42^ taking into account that the colors red and white have opposite biological meaning in the two scenarios. NIS-Elements AR software (Nikon Instruments, Melville, NY) was used to process the RGB images. The quadrant that included cortex, corpus callosum and hippocampus with normal-appearing brain surrounding the contusion was outlined manually. The hemorrhagic lesion area within this quadrant was determined, based on pixel intensities identified by a histogram of the green channel (dark pixels depict blood; brighter pixels depict normal brain). The percent hemorrhagic lesion was calculated based on the hemorrhagic lesion area and the area of the manually traced ipsilateral hemisphere. The percent hemispheric swelling was calculated based on the areas of the manually traced ipsilateral and contralateral hemispheres. This unbiased method of segmentation for determining the hemorrhagic lesion area was validated in a subset of images by two investigators (ZI, JAS), blinded to treatment group, who independently traced the hemorrhagic area manually on full color RGB images. HPC is defined as the increase in hemorrhagic lesion area at 24 h compared with 15 min after trauma.

### Immunolabeling and TUNEL staining of rat tissues

For rat tissues, cryosections were immunolabeled as above using the following primary antibodies: goat ant-SUR1 (1:500; custom antibody),^26^ chicken anti-TRPM4 (1:500; custom antibody), goat anti-TRPM4 (G-20; 1:200; sc-27540; Santa Cruz Biotechnology), or KIR6.2 (1:100; GTX80493; GeneTex, Irvine, CA). After labeling for one of the channel subunits, double immunolabeling was performed using either: CY3-conjugated, monoclonal anti-GFAP (1:500; C9205; Sigma-Aldrich), or monoclonal anti-RECA (MA1-81510; 1:100; HIS52; Invitrogen, ThermoFisher Scientific, Waltham, MA), or mouse anti-CD68 (ED1; 1:100; MAB1435; Millipore Sigma), to analyze cell specific expression. Secondary antibodies and subsequent image processing, as well as TUNEL labeling were performed as above.

### Statistical analysis

Data are presented as mean ± SEM, unless otherwise noted. The treatment effect (percent reduction in hemorrhagic lesion area or percent reduction in hemispheric swelling) was calculated as: (A_V_–A_D_)/(A_V_-A_B_)*100, where A_B_ is area at baseline, A_V_ is area at 24 h with vehicle/control treatment, and A_D_ is area at 24 h with drug treatment. Statistical analyses were performed using Origin Pro (V8; OriginLab Corp, Northampton, MA). Student's *t*-test and one-way analysis of variance with Tukey's honestly significant difference *post hoc* comparisons were used as appropriate.

## Results

**FIG. 2.**
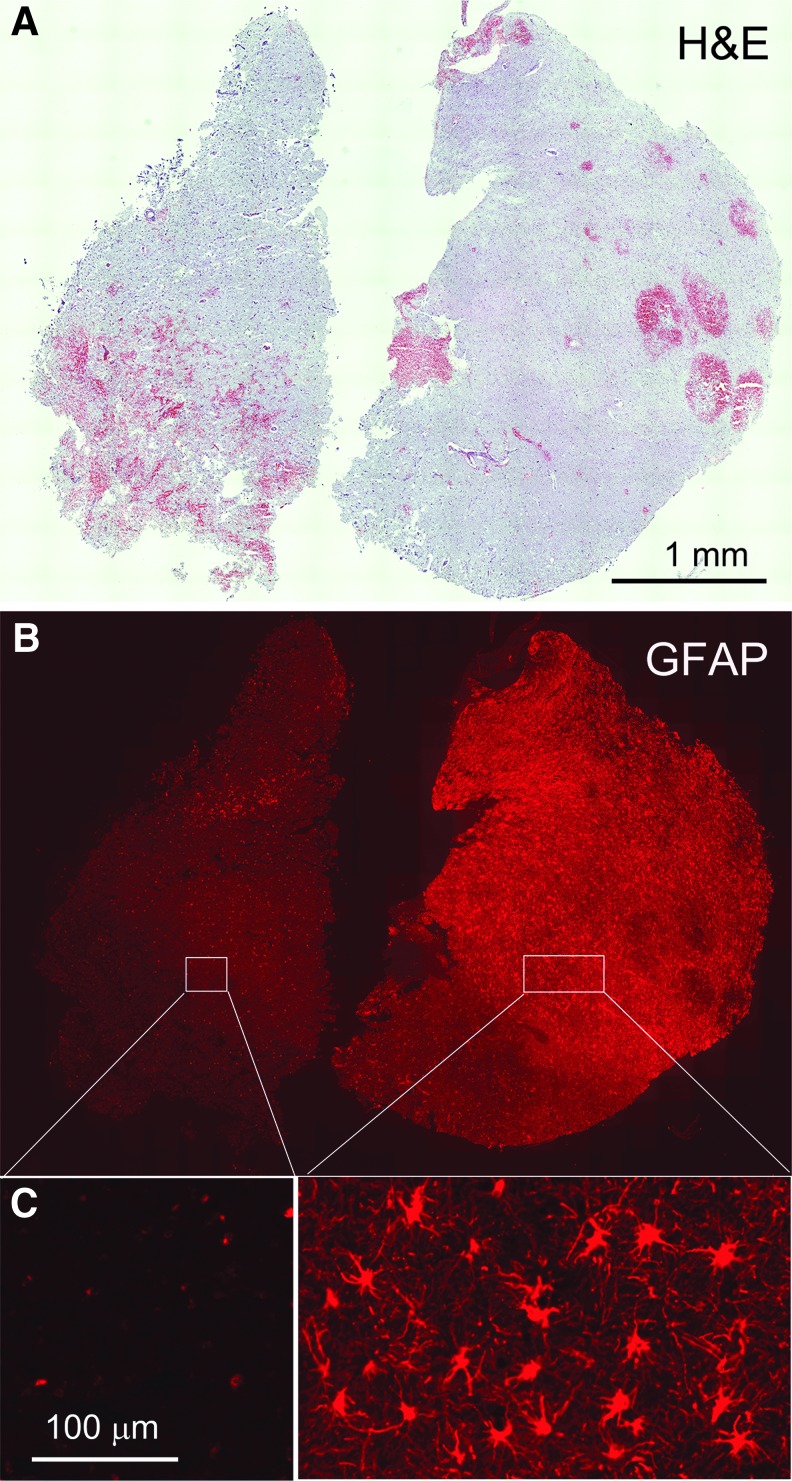
Glial fibrillary acidic protein (GFAP) immunoreactivity dichotomizes human contusion–traumatic brain injury (TBI) specimens. **(A-C)** Two sections from the same patient, stained with hematoxylin and eosin (A) or immunolabeled for GFAP (B, C), with the latter shown at low (B) and high (C) magnification: Note absence of specific immunoreactivity for GFAP in the specimen on the left vs. specific labeling of stellate-shaped cells in the companion specimen on the right. (case #2, 11 days post-TBI).

Brain tissue specimens from 16 patients with non-ballistic contusion-TBI who required decompressive craniectomy were first immunolabeled for GFAP. Since GFAP expression is reduced or absent in the core of ischemic and contusion injuries,^43–45^ whereas it is upregulated in surrounding penumbral regions, we used GFAP immunoreactivity to operationally classify our specimens as being GFAP-negative, likely from core regions, or GFAP-positive, likely from penumbral regions. In eight cases, GFAP labeling was absent or near-absent in all specimens, and in most of these, there was widespread TUNEL labeling indicative of severe cellular damage, consistent with these specimens originating from potentially non-viable core regions. In the other eight cases, GFAP immunolabeling was prominent in most of the specimens, with modest or no TUNEL labeling, consistent with these specimens originating from or containing viable penumbral tissues. In one of the GFAP-positive cases, two specimens were obtained, with one specimen showing robust GFAP immunoreactivity and the other specimen from the same patient being GFAP-negative (Fig. 2), illustrating the dichotomous nature of GFAP immunoreactivity in contusion specimens.

### GFAP-negative human specimens

**FIG. 3.**
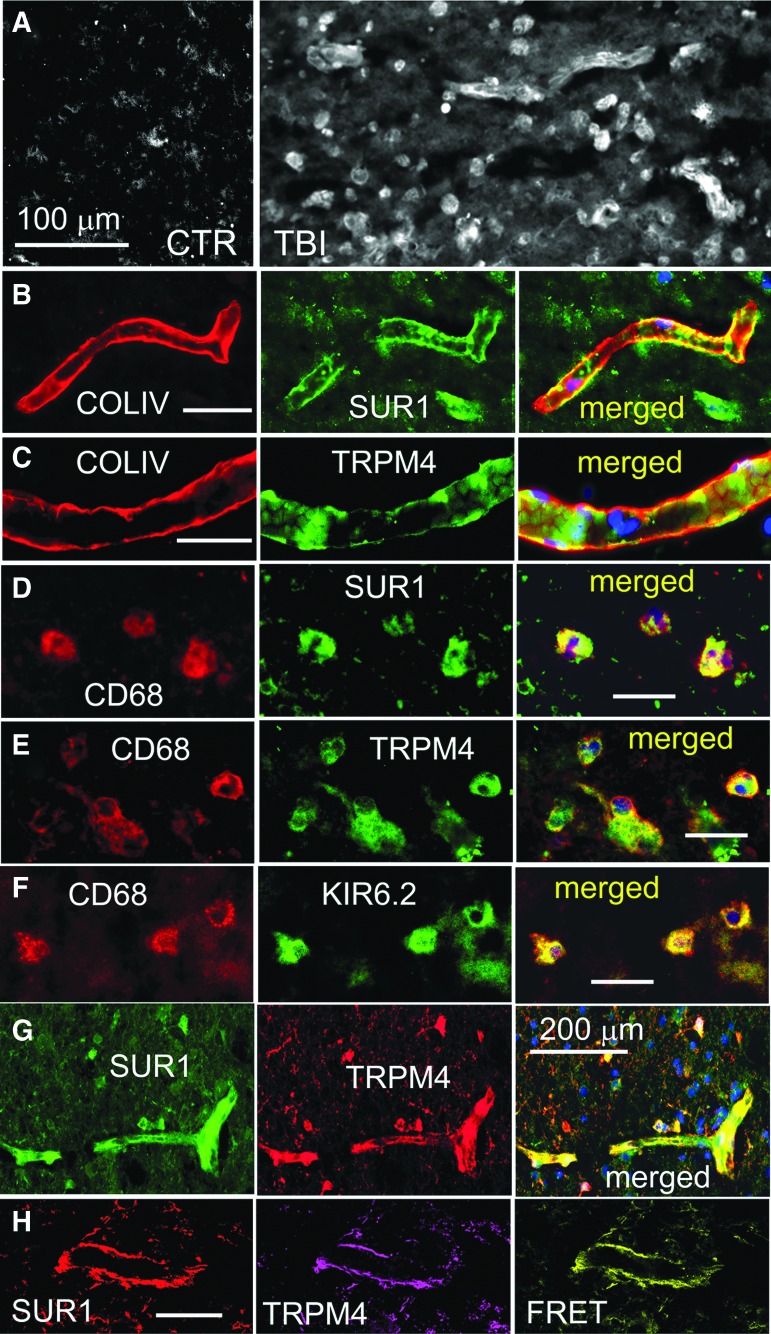
**(A)** Immunolabeling for sulfonylurea receptor 1 (SUR1) showed sparse immunoreactivity in the control specimen (CTR) vs. widespread expression in elongated structures and small round cells in a glial fibrillary acidic protein (GFAP)–negative specimen from contusion–traumatic brain injury (TBI). **(B, C)** Double immunolabeling for collagen IV (COLIV) (red) and SUR1 (B) or transient receptor potential cation channel subfamily M member 4 (TRPM4) (C) showed expression of SUR1 and TRPM4 in microvessels; merged images are also shown. **(D-F)** Double immunolabeling for CD68 (red) and SUR1 (D) or TRPM4 (E) or KIR6.2 (F) showed expression of SUR1, TRPM4 and KIR6.2 in microglia/macrophages; merged images are also shown. **(G)** Double immunolabeling for SUR1 (green) and TRPM4 (red) showed co-localization in a microvessel. **(H)** ImmunoFRET for SUR1 (red) and TRPM4 (magenta) shows co-assembly of SUR1-TRPM4 heteromers (yellow pseudocolor) in a microvessel. The findings illustrated are from the GFAP-negative specimen shown in Figure 2, left, and are representative of all GFAP-negative, SUR1-positive specimens from four cases of human contusion-TBI; (four cases with GFAP-negative specimens showed no immunoreactivity for SUR1). Case #2, 11 days post-TBI; case #3, 7 days post-TBI.

GFAP-negative specimens were immunolabeled for SUR1. Half of the GFAP-negative specimens also were negative for SUR1. The other GFAP-negative specimens showed prominent SUR1 expression, which was markedly greater compared with control tissues (Fig. 3A). Structures that were immunopositive for SUR1 consisted of elongated structures, likely microvessels, as well as small round cells (Fig. 3A).

Double immunolabeling for collagen IV, which identifies the basal lamina of microvessels, and for SUR1 or TRPM4 or KIR6.2, showed expression of SUR1 and TRPM4 in microvessels (Fig. 3B, 3C). Immunolabeling for CD68 showed that most of the small round cells were microglia/macrophages. Double immunolabeling for CD68 and SUR1 or TRPM4 or KIR6.2 showed expression of the three channel subunits in microglia/macrophages (Fig. 3D-F).^28^ Double immunolabeling showed that SUR1 and TRPM4 were co-localized in both microvessels and small round cells (Fig. 3G), and FRET imaging showed that SUR1 and TRPM4 co-assembled to form SUR1-TRPM4 heteromers in microvessels (Fig. 3H).

### GFAP-positive human specimens

**FIG. 4.**
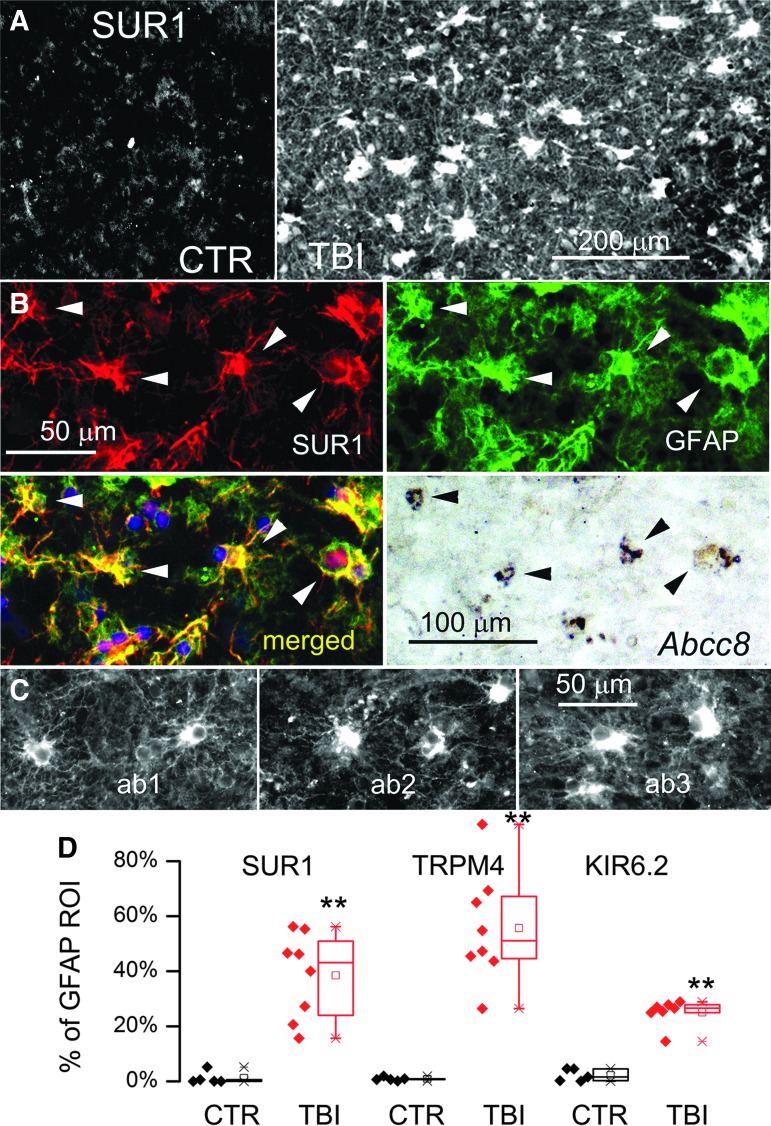
Glial fibrillary acidic protein (GFAP)–positive specimens from human contusion– traumatic brain injury (TBI) exhibit sulfonylurea receptor 1 (SUR1) expression in astrocytes. **(A)** Immunolabeling for SUR1 showed sparse immunoreactivity in the control specimen (CTR) vs. widespread expression in a GFAP-positive specimen from contusion-TBI. **(B)** Double immunolabeling for SUR1 (red) and GFAP (green) showed astrocyte expression of SUR1; merged images confirm co-localization (yellow); *in situ* hybridization of the same tissue section for *Abcc8* messenger RNA showed positive signal co-localized with GFAP-positive, SUR1-expressing astrocytes; arrowheads point to cells with all three signals. **(C)** Three adjacent tissue sections from a GFAP-positive specimen immunolabeled using three different anti-SUR1 antibodies: our custom polyclonal antibody^26^ (ab1), a monoclonal antibody (S289-16; Novus Biologicals; ab2), and a commercial polyclonal antibody (sc-5789; Santa Cruz Biotechnology; ab3): Note comparable labeling by all three antibodies of stellate-shaped cells with good signal-to-noise ratio and minimal background labeling (case #2, 11 days post-TBI; case #5, 1 day post-TBI). **(D)** Quantification of SUR1, transient receptor potential cation channel subfamily M member 4 (TRPM4), and KIR6.2 expression in GFAP-positive astrocytes in TBI specimens (red) vs. controls (black); quantification performed based on GFAP-defined region of interest; scatterplots as well as box plots are shown; each symbol is from a different case; box plot symbols: small box, mean; ––, median; large box, 25th and 75th percentile; × , 1st and 99th percentile; −, max and min: Note that in controls, GFAP-positive astrocytes expressed minimal channel subunits, whereas all three channel subunits were overexpressed in GFAP-positive astrocytes in contusion-TBI specimens; **p < 0.01. The findings illustrated are representative of all GFAP-positive specimens from eight cases of human contusion-TBI.

GFAP-positive specimens were immunolabeled for SUR1. All of the GFAP-positive specimens also were positive for SUR1, which was markedly greater compared with control tissues (Fig. 4A), as reported.^13^ The most prominent structures that were immunopositive for SUR1 were stellate-shaped cells (Fig. 4A). Double immunolabeling for SUR1 and GFAP or CD68 or collagen IV showed that SUR1 expression was by far more prominent in astrocytes and minimal or absent in microvessels or microglia/macrophages (Fig. 4B). SUR1 expression in occasional non-GFAP-positive cells, some of which may have been neurons or microglia/macrophages,^13^ was not studied in these specimens.

GFAP-positive cells that expressed SUR1 protein also expressed *ABCC8* mRNA (Fig. 4B). Immunolabeling with two other anti-SUR1 antibodies, a monoclonal (S289-16; Novus Biologicals) and a commercial polyclonal (sc-5789; Santa Cruz Biotechnology), yielded images similar to those obtained with the custom anti-SUR1 antibody (Fig. 4C). Quantification using GFAP as the region of interest confirmed that SUR1 was significantly upregulated in penumbral astrocytes post-TBI (Fig. 4D).

**FIG. 5.**
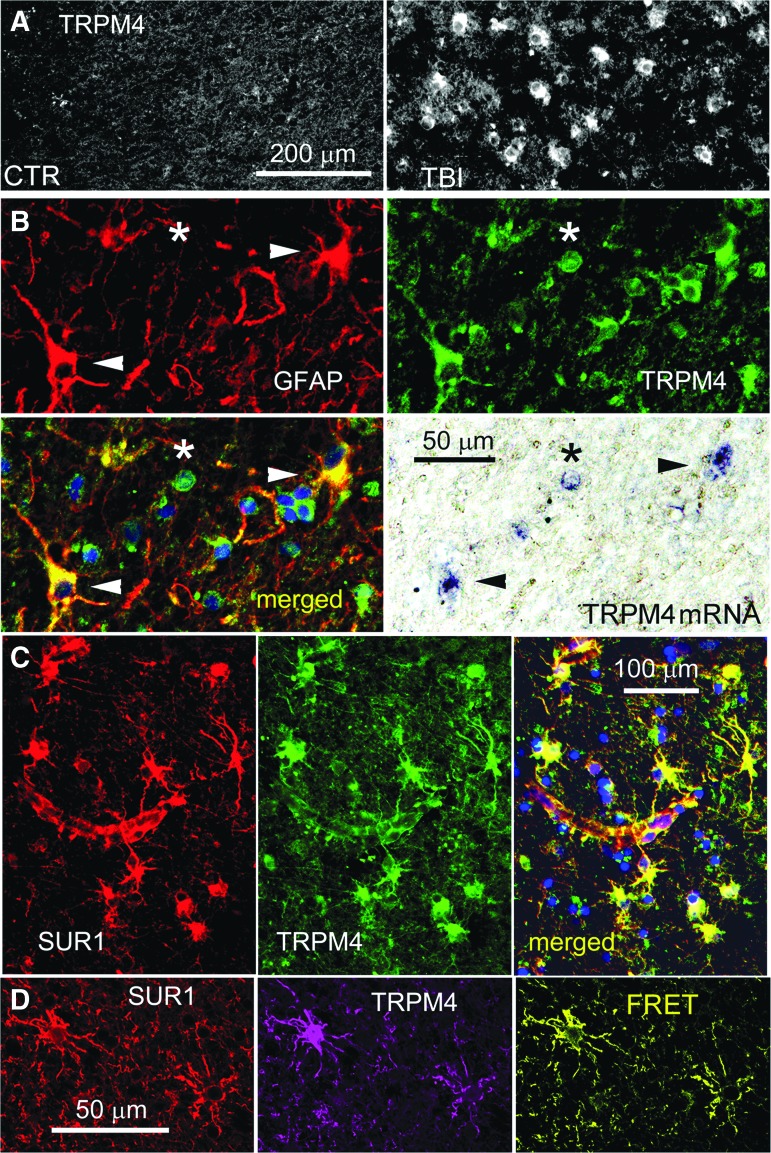
Glial fibrillary acidic protein (GFAP)–positive specimens from human contusion– traumatic brain injury (TBI) exhibit transient receptor potential cation channel subfamily M member 4 (TRPM4) expression in astrocytes. **(A)** Immunolabeling for TRPM4 showed sparse immunoreactivity in the control specimen (CTR) vs. widespread expression in a GFAP-positive specimen from contusion-TBI (TBI). **(B)** Double immunolabeling for GFAP (red) and TRPM4 (green) showed astrocyte expression of TRPM4; merged images confirms co-localization (yellow); *in situ* hybridization of the same tissue section for *Trpm4* messenger RNA showed positive signal co-localized with GFAP-positive, TRPM4-expressing astrocytes; arrowheads point to stellate-shaped cells with all three signals; asterisk denotes GFAP-negative small round cell with TRPM4 protein and mRNA. **(C)** Double immunolabeling showing sulfonylurea receptor 1 (SUR1; red) and TRPM4 (green) co-localization in perivascular astrocytes; merged images also shown (yellow). **(D)** ImmunoFRET for SUR1 (red) and TRPM4 (magenta) shows co-assembly of SUR1-TRPM4 heteromers (yellow pseudocolor) in astrocytes. The findings illustrated are representative of all GFAP-positive specimens from eight cases of human contusion-TBI. (case #2, 11 days post-TBI; case #5, 1 day post-TBI)

Like SUR1, TRPM4 was minimally expressed in control cortical tissues, but was upregulated in GFAP-positive penumbral specimens (Fig. 5A), similar to that reported in diffuse TBI.^27^ Double immunolabeling with GFAP showed that TRPM4 expression was prominent in astrocytes, and could also be observed in small round cells (Fig. 5B). GFAP-positive cells that expressed TRPM4 protein also expressed *TRPM4* mRNA, as did some small round cells (Fig. 5B). Co-immunolabeling showed that astrocytes that labeled positive for TRPM4 also labeled positive for SUR1 (Fig. 5C), and FRET experiments showed that TRPM4 and SUR1 formed heteromers of SUR1-TRPM4 (Fig. 5D). Quantification using GFAP as the region of interest confirmed that TRPM4 was significantly upregulated in cortical astrocytes post-TBI (Fig. 4D).

**FIG. 6.**
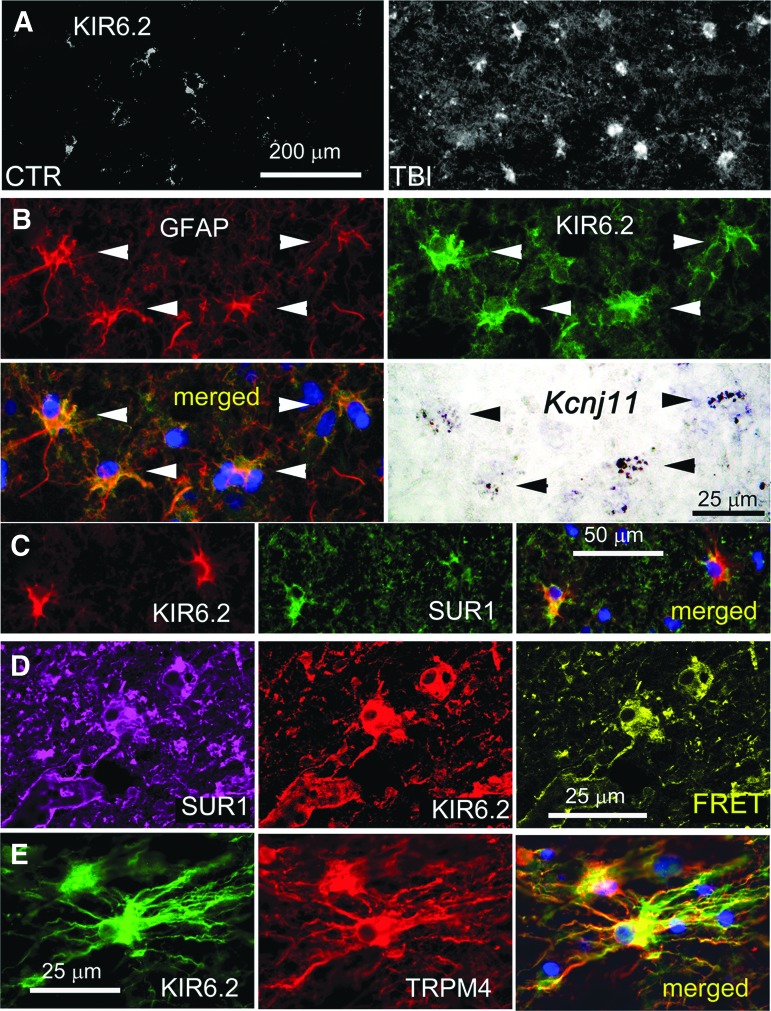
Glial fibrillary acidic protein (GFAP)–positive specimens from human contusion- traumatic brain injury (TBI) exhibit KIR6.2 expression in astrocytes. **(A)** Immunolabeling for KIR6.2 showed sparse immunoreactivity in the control specimen (CTR) vs. widespread expression in a GFAP-positive specimen from contusion-TBI. **(B)** Double immunolabeling for GFAP (red) and KIR6.2 (green) showed astrocyte expression of KIR6.2; merged images confirm co-localization (yellow); *in situ* hybridization of the same tissue section for *Kcnj11* messenger RNA showed positive signal co-localized with GFAP-positive, KIR6.2-expressing astrocytes; arrowheads point to cells with all three signals. **(C)** Double immunolabeling showed that KIR6.2 (red) and sulfonylurea receptor 1 (SUR1; green) were co-localized (yellow) in astrocytes. **(D)** ImmunoFRET for SUR1 (magenta) and KIR6.2 (red) showed co-assembly of SUR1-KIR6.2 heteromers (yellow pseudocolor) in astrocytes. **(E)** Double immunolabeling showed that KIR6.2 (green) and transient receptor potential cation channel subfamily M member 4 (TRPM4) (red) were co-localized in astrocytes. The findings illustrated are representative of all GFAP-positive specimens from eight cases of human contusion-TBI. (case #2, 11 days post-TBI; case #5, 1 day post-TBI)

Like SUR1 and TRPM4, KIR6.2 was minimally expressed in control cortical tissues, except in occasional neurons, but was upregulated in GFAP-positive penumbral specimens (Fig. 6A), as reported.^28^ Double immunolabeling with GFAP showed that KIR6.2 expression was prominent in astrocytes (Fig. 6B). KIR6.2 expression in non-GFAP-positive cells, presumably neurons, was sparse and did not differ appreciably from controls.^28^ GFAP-positive cells that expressed KIR6.2 protein also expressed KCNJ11 mRNA (Fig. 6B). Co-immunolabeling showed that cells that labeled positive for KIR6.2 also labeled positive for SUR1 (Fig. 6C), and FRET experiments showed that KIR6.2 and SUR1 formed heteromers of SUR1-KIR6.2 (Fig. 6D). Quantification using GFAP as the region of interest confirmed that KIR6.2 was significantly upregulated in cortical astrocytes post-TBI (Fig. 4D).

Double immunolabeling showed that TRPM4 and KIR6.2 were expressed in the same cells whose stellate morphology was consistent with astrocytes (Fig. 6E). Overall, qualitative assessment of the eight GFAP-positive specimens revealed that the three subunits, SUR1, TRPM4, and KIR6.2, tended to be upregulated in parallel in astrocytes, that expression during the first 24 h after injury tended to be variable, and that later, essentially all astrocytes upregulated all three subunits.

### Channel subunit expression in rat contusion-TBI

**FIG. 7.**
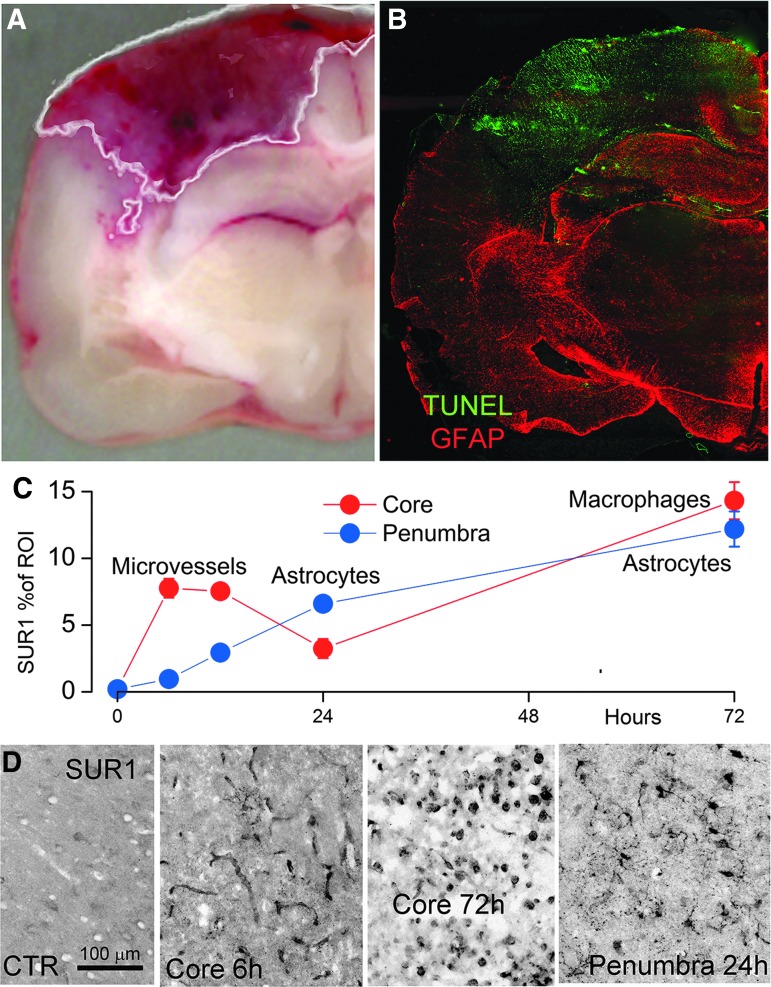
Sulfonylurea receptor 1 (SUR1) expression in rat contusion- traumatic brain injury (TBI). **(A, B)** Coronal image of contusion (A) and coronal section immunolabeled for glial fibrillary acidic protein (GFAP; red) and stained for TUNEL (green; B), showing that the hemorrhagic core is largely devoid of GFAP immunoreactivity, but shows widespread TUNEL labeling; the white line in (A) depicts the output of the algorithm-based segmentation protocol used to identify the hemorrhagic lesion area (see Methods). (**C, D)** Quantification of total SUR1 expression in core (red) vs. penumbra (blue) as a function of time post-contusion-TBI; five rats per time-point; the labels “microvessels,” “astrocytes,” and “macrophages” in (C) are based on high magnification views (D), showing that: 1) early in the core, SUR1 is most prominent in elongated structures consistent with microvessels; 2) later in the core, SUR1 is most prominent in small round cells; and 3) at all times in the penumbra, SUR1 is most prominent in stellate cells consistent with astrocytes.

In the rat contusion model, the lesion core identified by the hemorrhagic area in unstained sections showed markedly reduced GFAP immunoreactivity^45^ along with markedly elevated TUNEL labeling (Fig. 7A, 7B), consistent with previous findings,^43–45^ and consistent with our rational for dichotomizing the human specimens.

We studied SUR1 expression in the core and penumbra as a function of time. In the core, SUR1 expression was significantly increased over controls, it peaked at 6 h, remained elevated at 12 h, declined at 24 h, then rose again at 72 h (Fig. 7C).^4,12^ During the first 24 h, the primary structures within the core that labeled for SUR1 were elongated structures, consistent with microvessels (Fig. 7D). However, at 72 h, labeling of microvessels was no longer evident and instead, labeling was most prominent in small round cells (Fig. 7D). SUR1 immunolabeling of the GFAP-negative core did not show any stellate-shaped cells. By contrast, SUR1 immunolabeling of the GFAP-positive penumbra showed numerous stellate-shaped cells, with these cells being the predominant cell-type (Fig. 7D).

**FIG. 8.**
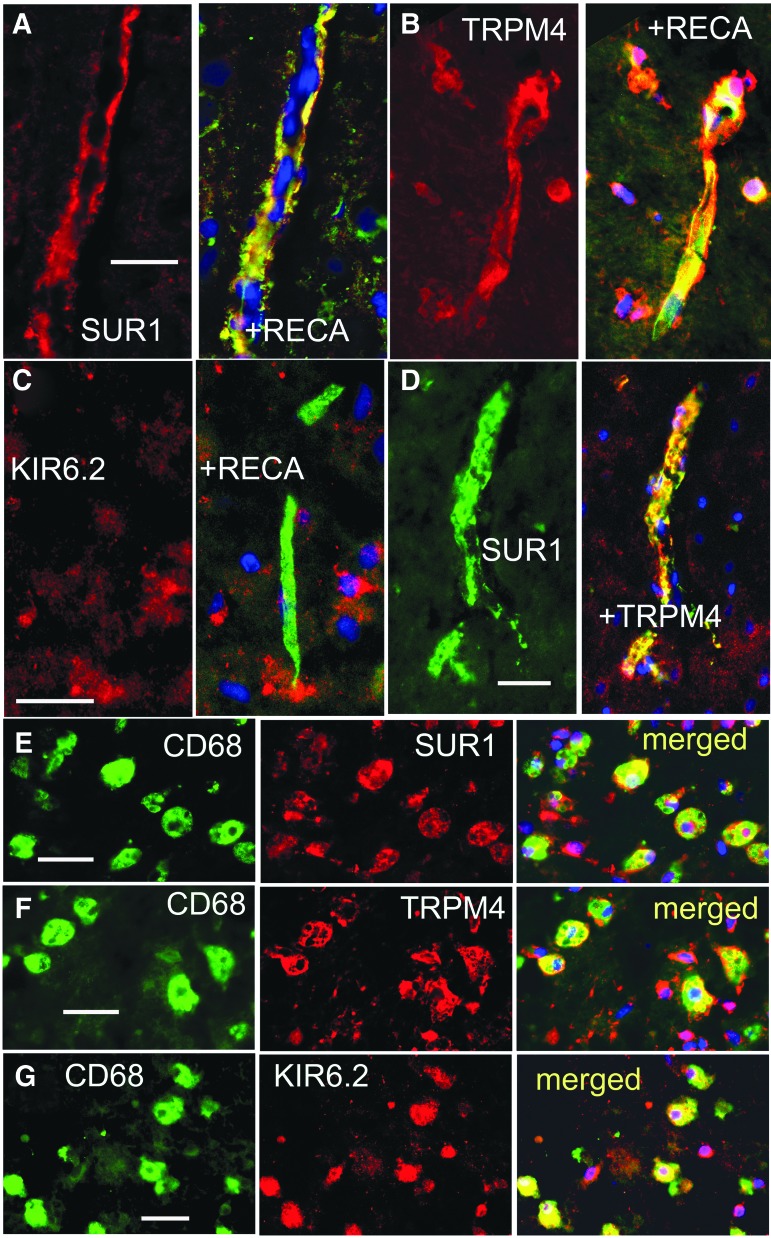
Glial fibrillary acidic protein (GFAP)–negative specimens from rat contusion–traumatic brain injury (TBI) exhibit sulfonylurea receptor 1 (SUR1) expression in microvessels and microglia/macrophages. **(A-C)** Double immunolabeling for rat endothelial cell antigen (RECA) (green) and SUR1 (A) or transient receptor potential cation channel subfamily M member 4 (TRPM4) (B) or KIR6.2 (C) at 24 h post-TBI showed expression of SUR1 and TRPM4 in microvessels; merged images are shown in the right panels (yellow). **(D)** Double immunolabeling for SUR1 (green) and TRPM4 (red) at 24 h post-TBI shows co-localization in microvessels. **(E-G)** Double immunolabeling for CD68 (green) and SUR1 (E) or TRPM4 (F) or KIR6.2 (G) at 72 h post-TBI showed expression of SUR1, TRPM4, and KIR6.2 in microglia/macrophages; merged images are also shown. The findings illustrated are representative of five specimens from rat contusion-TBI with GFAP-negative core.

In core tissues, double immunolabeling with RECA and SUR1 or TRPM4 or KIR6.2 showed microvascular expression of SUR1 and TRPM4, but no convincing evidence of KIR6.2 (Fig. 8A-C). Double immunolabeling of core tissues showed that SUR1 and TRPM4 were co-localized in microvessels (Fig. 8D).

In core tissues, double immunolabeling with CD68 and SUR1 or TRPM4 or KIR6.2 showed that microglia/macrophages accounted for most of the small round cells, and that these cells expressed all three channel subunits (Fig. 8E-G).

**FIG. 9.**
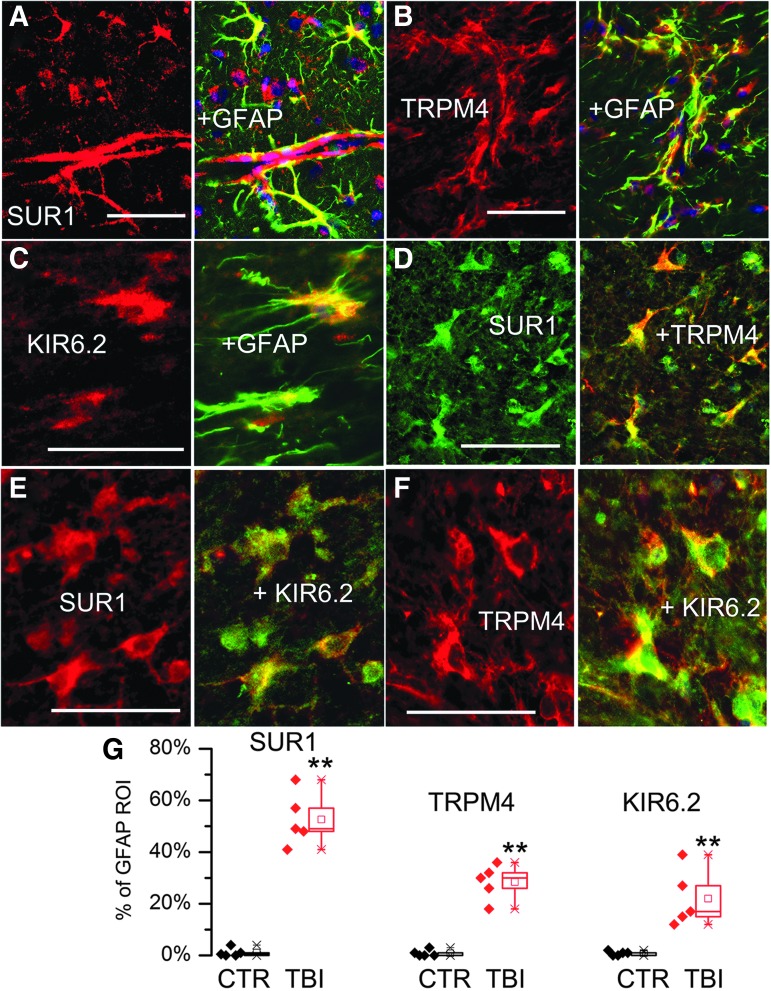
Glial fibrillary acidic protein (GFAP)–positive specimens from rat contusion– traumatic brain injury (TBI) exhibit sulfonylurea receptor 1 (SUR1) expression in astrocytes. **(A-C)** Double immunolabeling for GFAP (green) and SUR1 (A) or transient receptor potential cation channel subfamily M member 4 (TRPM4) (B) or KIR6.2 (C) showed expression of all three channel subunits in astrocytes; merged images are shown in the right panels (yellow). **(D-F)** Double immunolabeling for SUR1 and TRPM4 (D), SUR1 and KIR6.2 (E), and TRPM4 and KIR6.2 (F), shows co-localization in astrocytes; merged images are shown in the right panels (yellow). **(G)** Expression of SUR1, TRPM4 and KIR6.2 in GFAP-positive astrocytes in TBI specimens (red) vs. controls (black); quantification performed based on GFAP-defined region of interest; scatterplots as well as box plots are shown; box plot symbols are the same as in Figure 4: Note that in controls, GFAP-positive astrocytes expressed minimal channel subunits, whereas all three channel subunits were expressed in GFAP-positive astrocytes in contusion-TBI specimens; **p < 0.01.

In the penumbra, double immunolabeling with GFAP and SUR1 or TRPM4 or KIR6.2 showed that astrocytes expressed all three channel subunits (Fig. 9A-C). Quantification using GFAP as the region of interest confirmed that SUR1, TRPM4 and KIR6.2 were significantly upregulated in penumbral astrocytes post-TBI (Fig. 9G). Double immunolabeling showed that SUR1 and TRPM4, SUR1 and KIR6.2, as well as TRPM4 and KIR6.2 all co-localized in astrocytes (Fig. 9D-F).

### Glibenclamide and HPC

**FIG. 10.**
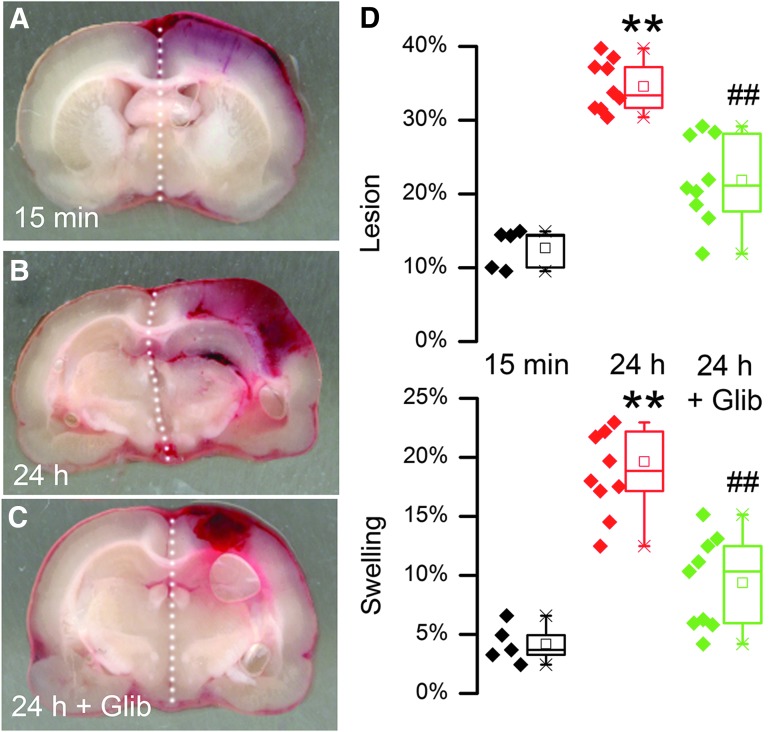
Glibenclamide reduces hemorrhagic progression of contusion (HPC). **(A-C)** Images of coronal sections of brains through the epicenter, obtained in vehicle-treated animals 15 min after trauma (A) and 24 h after trauma (B), or in a glibenclamide-treated animal 24 h after trauma (C); the increase in area of brain exhibiting hemorrhagic transformation between 15 min and 24 h represents HPC. **(D)** Quantification of hemorrhagic lesion area (above) or hemispheric swelling (below) in vehicle-treated animals 15 min after trauma (black) and 24 h after trauma (red), or in glibenclamide-treated animals 24 h after trauma (green); 5 or 9 rats per group; box plot symbols same as in Figure 4; **p < 0.01 with respect to 15 min; ##*p* < 0.01 with respect to 24 h controls.

In the rat model, quantification of the hemorrhagic lesion area at baseline (15 min) versus 24 h was used to gauge HPC. At baseline, the hemorrhagic lesion area, reflecting the primary hemorrhage, was small, whereas by 24 h, the hemorrhagic lesion area had grown significantly (Fig. 10), reflecting the fact that the primary hemorrhage had undergone HPC.^4^ Images of the hemorrhages at 24 h confirmed that the increase in blood was not due to an increase in bleeding from a point source, but was dispersed throughout the tissues, consistent with hemorrhagic transformation of brain (Fig. 10B). Measurements of hemispheric volume showed that hemispheric swelling was commensurate with hemorrhagic lesion area (Fig. 10D). Glibenclamide treatment initiated within 15 min after trauma yielded a hemorrhagic lesion area at 24 h that was significantly less than in vehicle-treated animals, reflecting a reduction in mean HPC by ∼58%. Similarly, glibenclamide treatment was associated with hemispheric swelling at 24 h that was significantly less than in vehicle-treated animals, with a reduction in mean swelling of ∼66%.

### AS-ODN and HPC

We used AS-ODNs to assess the functional role of the three channel subunits in HPC. Circulating intravascular oligonucleotides normally are excluded from the brain parenchyma by the intact blood–brain barrier (BBB),^46^ but TBI causes BBB disruption^47^ that enables delivery of circulating oligonucleotides to brain cells. Moreover, following central nervous system (CNS) trauma, AS-ODNs may be selectively taken up by activated endothelium.^39,48^

**FIG. 11.**
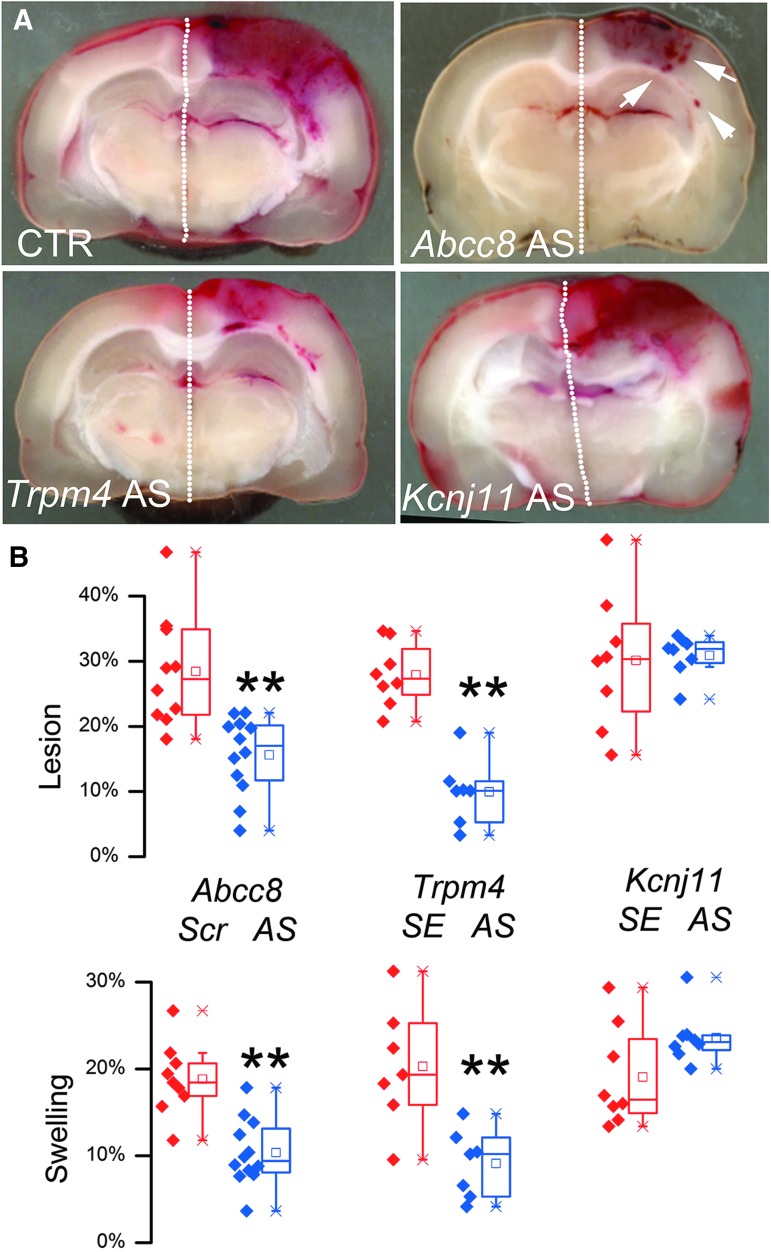
AS-ODN targeting *Abcc8* and *Trpm4*, but not *Kcnj11*, reduces hemorrhagic progression of contusion (HPC). **(A)** Images of coronal sections of brains through the epicenter, obtained 24 h after trauma in animals administered control-ODN (CTR), *Abcc8*-ODN, *Trpm4*-ODN or *Kcnj11*-ODN, as indicated; arrowheads point to distinct petechial hemorrhages. **(B)** Quantification of hemorrhagic lesion area (above) or hemispheric swelling (below), in control-ODN-treated animals 24 h after trauma (red), and in *Abcc8*- or *Trpm4*- or *Kcnj11*-ODN-treated animals 24 h after trauma (blue); control-ODN was either scrambled (Scr) or sense (SE) ODN, as indicated; 7–12 rats per group; box plot symbols same as in Figure 4; **p < 0.01 with respect to control; analysis of variance revealed no statistically significant difference between the three controls, or between *Kcnj11*-ODN-treated animals and controls.

Within 15 min of trauma, rats were administered intravenous AS-ODNs directed against *Abcc8*, *Trpm4,* or *Kir6.2* mRNA. The hemorrhagic lesion areas in controls for the three treatments were not significantly different. Compared with controls, anti-*Abcc8* and anti-*Trpm4* AS-ODNs significantly reduced hemorrhagic lesion areas at 24 h, while values for the two treatments were not significantly different from each other (Fig. 11). Assuming baseline values comparable to those above, mean HPC was reduced ∼70% and ∼92% by anti-*Abcc8* and anti-*Trpm4* AS-ODNs, respectively. Similarly, AS-ODN treatment was associated with hemispheric swelling at 24 h that was significantly less than in controls (Fig. 11), with reductions in mean swelling of ∼58% and ∼70% by anti-*Abcc8* and anti-*Trpm4* AS-ODNs, respectively. By contrast, following administration of anti-*Kir6.2* AS-ODN, hemorrhagic lesion area and hemispheric swelling at 24 h were not significantly different compared with controls (Fig. 11).

## Discussion

There are two major findings in the present report. First, individual subunits and heteromers of SUR1-TRPM4 and K_ATP_ (SUR1-KIR6.2) channels were upregulated after human TBI, confirming and expanding on recent reports on SUR1 and KIR6.2 expression in human TBI,^13,28^ and recapitulating observations on SUR1 and TRPM4 in animal models of contusion-TBI.^4,12,27^ Second, our data with AS-ODNs in the rat contusion model showed that SUR1-TRPM4, not K_ATP_ (SUR1-KIR6.2), accounts best for post-contusion HPC. Overall, our data support the hypotheses that SUR1-TRPM4 plays a crucial role in contusion-TBI, and that a primary target of glibenclamide in TBI is SUR1-TRPM4.

### Channel subunit expression in human contusions

Dichotomizing tissues as being GFAP-positive versus GFAP-negative facilitated our investigation of channel subunit expression in various cell types. This classification is justified by observations both here and previously that GFAP expression is reduced or absent in the core of ischemic and contusion injuries,^43–45^ whereas it is upregulated in surrounding penumbral regions. Equating GFAP immunoreactivity with core versus penumbra may be a broad generalization, but it was useful for predicting the cell types and patterns of SUR1 expression.

In GFAP-negative core tissues, SUR1 was upregulated most prominently in two cell types, microvascular endothelium and CD68-positive microglia/macrophages. By contrast, in GFAP-positive penumbral tissues, SUR1 was upregulated predominantly in astrocytes. In specimens characterized using FRET, we found evidence of SUR1-TRPM4 heteromers in microvascular endothelium, whereas both SUR1-TRPM4 and SUR1-KIR6.2 heteromers were identified in astrocytes. Cortical neurons also express SUR1 as part of K_ATP_ channels, and SUR1 in neurons previously was reported to be increased in human TBI specimens,^13^ but here, we did not quantify channel subunit expression in neurons.

Overall, our findings in human TBI accord with observations on SUR1 in human TBI by Martinez-Valverde and colleagues,^13^ and they accord with findings on SUR1 in TBI rat models reported here and previously.^4,12^ Also, our findings on KIR6.2 accord with previous observations in human TBI by Castro and colleagues,^28^ who first reported upregulation of KIR6.2, predominantly in astrocytes. The present study is the first to report on TRPM4 in the human brain after TBI, and our findings are in accord with those reported in a diffuse-TBI rat model.^27^

### K_ATP_

K_ATP_ channels are activated by processes associated with energy deprivation (e.g., a fall in the ATP/ADP ratio). Work investigating the function of neuronal K_ATP_ channels under pathological conditions has focused primarily on their role in hypoxia or excitotoxicity.^49–51^ These studies showed that K_ATP_ channels mediate neuronal hyperpolarization, which counteracts anoxic/ischemic depolarization, and thereby reduces neuronal death. In accord with this, KIR6.2-null mice are vulnerable to brain hypoxia, and exhibit a reduced threshold for hypoxia-induced generalized seizures,^52,53^ while KIR6.2-overexpressing mice are protected.^54^

Whereas constitutive KIR6.2 expression by neurons is established, KIR6.2 expression in glial cells in the normal brain is controversial, or may depend on location. Dunn-Meynell and colleagues^55^ reported that while various neurons in rat brain express KIR6.2 mRNA, neither astrocytes nor oligodendrocytes express KIR6.2 mRNA. Conversely, Zhou and colleagues^56^ showed that glia in the rodent corpus callosum and cerebellar white matter (but not cerebral cortex) weakly express KIR6.2. In the present study, as well as in the report by Castro and colleagues,^28^ KIR6.2 was not identified in astrocytes in control human cortex.

Conversely, several reports indicate that astrocytes normally express the closely related subunit KIR6.1.^57,58^ Moreover, recent data indicate that in disease states, there may be a dramatic increase in KIR6.2 expression in reactive protoplasmic and fibrous astrocytes, whereas no such increase is observed for KIR6.1, a phenomenon that was termed “aberrant expression of KIR6.2.”^59^ In both the report by Castro and colleagues^28^ as well as here, so-called aberrant expression of KIR6.2 in reactive astrocytes was identified post-TBI. Although questions may remain about constitutive expression of KIR6.2 in quiescent astrocytes, upregulation of KIR6.2 in cortical reactive astrocytes under pathological conditions seems certain.

In contrast to neurons and astrocytes, K_ATP_ channel subunits are essentially undetectable in endothelium of normal brain microvessels or endothelium of the basilar and middle cerebral arteries, although smooth muscle cells of cerebral arteries express the various subunits.^60,61^ Vascular smooth muscle K_ATP_ channels mediate vasodilation in response to hypoxia.^62,63^

The role of K_ATP_ in TBI, especially in penumbral astrocytes where KIR6.2 is strongly upregulated, remains unknown. Our experiments with AS-ODN targeting KIR6.2 did not identify a role for KIR6.2, and by inference for K_ATP_, in brain swelling or HPC. Given the prominent expression of K_ATP_ in penumbral astrocytes post-TBI, we infer that astrocytes do not play a substantive role in HPC. Although we cannot rule out a problem with AS-ODN entry into astrocytes *in vivo* in our experiments, we note that the same AS-ODN was shown to be effective in downregulating KIR6.2 in basal ganglia neurons *in vivo.*^40^ Importantly, our negative data with anti-KIR6.2 AS-ODN do not imply that KIR6.2 upregulation in reactive astrocytes is without function, only that our experiments did not identify that function in TBI. Future work will examine this important question more carefully.

### SUR1-TRPM4

In animal models of contusion-TBI, blockade of SUR1 with glibenclamide reduces capillary fragmentation in the penumbra, which is the immediate cause of HPC.^4^ In our previous study, we quantified HPC based on spectrophotometric analysis of tissue hemoglobin, and found that the hemoglobin content increased ∼2-fold between baseline and 24 h, and that this increase was blunted by ∼90% by glibenclamide.^4^ In the present study, we quantified HPC based on the area of hemorrhagic brain visible on single coronal images for each rat, and found that the hemorrhagic area increased ∼2-fold between baseline and 24 h, and that this increase was blunted by ∼50% by glibenclamide. Independent studies in rat and mouse models of contusion-TBI that used either MRI^14^ or tissue hemoglobin^15^ reported that glibenclamide reduced hemorrhagic lesion volumes by ∼50%. A study in humans with contusion-TBI that used volumetric analysis of CT scans found that glibenclamide reduced hemorrhagic lesion volumes by ∼80%.^22^ This broad concordance on the robust effect of glibenclamide across independent laboratories and across different species underscores the important role of SUR1 in HPC.

Glibenclamide is a sulfonylurea drug that targets sulfonylurea receptors, of which three are known: SUR1, SUR2A, and SUR2B. Of these, glibenclamide exhibits the highest potency at SUR1.^64^ Here, we found that the effects of glibenclamide on HPC and brain swelling were closely mimicked by anti-SUR1 AS-ODN, which largely eliminates involvement of SUR2A and SUR2B in HPC after contusion-TBI, and is consistent with glibenclamide acting via SUR1, not via some off-target effect.

We showed that the effects of blocking SUR1 are replicated using AS-ODN directed against *Trpm4*. These findings establish, for the first time, that SUR1-TRPM4 is a dominant molecular mechanism responsible for HPC. Notably, previous work in non-TBI models also showed that suppression of SUR1 and TRPM4 results in an essentially identical post-injury phenotype. In a contusion spinal cord injury model, genetic suppression of either subunit is similarly effective at preventing “progressive hemorrhagic necrosis,”^39,48,65^ a process of hemorrhagic lesion expansion in SCI that is similar to HPC in TBI. In experimental allergic encephalomyelitis, genetic suppression of either subunit is similarly effective at reducing inflammation and disease progression.^66^ In activated microglia *in vitro*, pharmacological inhibition of either subunit is similarly effective at reducing transcription of nitric oxide synthase 2.^67^ In activated astrocytes *in vitro,* pharmacological inhibition of either subunit is similarly effective at reducing cell swelling.^68^ Thus, in diverse CNS injury scenarios, the SUR1-TRPM4 channel plays a critical role in pathogenesis, with blockade or inhibition of either subunit yielding a similar favorable post-injury phenotype.

### Co-expression of K_ATP_ and SUR1-TRPM4

Some cells, including astrocytes and microglia/macrophages, were found to express both K_ATP_ and SUR1-TRPM4 channels. As plasmalemmal channels, these two channels have opposite effects on membrane polarization—upon activation, K_ATP_ channels mediate K^+^ efflux and cell hyperpolarization, whereas SUR1-TRPM4 channels mediate Na^+^ influx and cell depolarization. These opposite functions may not conflict, however, since the two channels are regulated differently by intracellular ATP and calcium, which interact with the pore-forming subunit, not SUR1. K_ATP_ (SUR1-KIR6.2) is inhibited by ATP with an EC_50_ ∼18 μM, and the inhibitory action of ATP is counteracted by ADP,^69,70^ whereas SUR1-TRPM4 is inhibited by ATP with an EC_50_ 0.8 μM, with no effect of ADP.^71^ Moreover, of the two subunits, only TRPM4 is sensitive to intracellular calcium/calmodulin.^26,71^ Thus, although both have the same SUR1 regulatory subunit, independent regulation of the two channels via the different pore-forming subunits is likely. Other cells also are known to express SUR1, KIR6.2, and TRPM4 simultaneously, including pancreatic β cells^72^ and neurons of the arcuate nucleus.^73^ Notably, in pancreatic β cells, K_ATP_ and TRPM4 function together to regulate insulin secretion,^72,74^ illustrating co-operative, not mutually opposed, functions. Further study is needed to determine the subcellular localization and to identify the complex functional interactions served by co-expression of K_ATP_ and SUR1-TRPM4 in reactive astrocytes and microglia/macrophages post-TBI.

### Brain swelling

In TBI, brain swelling is directly responsible for major morbidity and death.^10,11^ In TBI, more so than in ischemia, it is important to distinguish between “brain swelling” and “brain edema,” two terms that are occasionally conflated but are not equivalent. Whereas edema contributes to brain swelling, edema is only one of several distinct space-occupying components responsible for swelling. Brain swelling arises from the combined space-occupying effects of extravasated blood, extracellular edema fluid, cellular swelling, vascular engorgement, and, possibly, hydrocephalus. Extravasated blood from both the initial contusion and from HPC contributes in a major way to brain swelling. Extracellular edema fluid, which develops later via two distinct mechanisms (see below), is another important contributor. Cellular swelling, especially of astrocytes, may be a larger contributor to brain swelling than is usually acknowledged.^27,75^ Post-traumatic vascular engorgement or hyperemia, which is attributed to vasoparalysis or venous outflow obstruction, also may contribute significantly to brain swelling.^76,77^ Some of these processes develop rapidly, while others worsen progressively over days. All have different underlying mechanisms, and while some may be inhibitable, at least in principal, others are not.

In the present study, extravasated blood was found to be a major contributor to brain swelling. The finding that glibenclamide, anti-SUR1 AS-ODN and anti-TRPM4 AS-ODN reduced brain swelling at 24 h by approximately half (Fig. 10 and Fig. 11) is best attributed to a treatment-related reduction in secondary hemorrhage from HPC, which was of a similar magnitude. At 24 h, extravasated blood seems to be the dominant factor responsible for brain swelling in this TBI model. This finding accords with the observation in humans that midline shift, a measure of hemispheric swelling, and hematoma volume are nearly co-linear.^10^ Notably, pharmacological inhibition of SUR1 was shown recently in a human clinical trial to reduce HPC after TBI.^22^

In TBI, edema forms via two distinct mechanisms, with the distinction based on the origin of the osmotically active solute, either intracontusional or intravascular. The intracontusional component was characterized by Katayama and colleagues,^78^ who showed that hemorrhagic/necrotic lesions in BCs have very high osmolarity. The increase in osmotic pressure is due to metabolic production of osmoles or the release of idiogenic osmoles from necrotic tissues.^79^ The high osmolarity of the hyperosmolar core acts as a powerful force that attracts water from the perilesional brain through a compromised BBB. In this case, the causative osmolytes originate from within the contusion. This component of edema can be reduced by intravascular osmotherapies, which counteract the transport of water from the vascular compartment into the tissues, but because water flux from the vascular compartment cannot be specifically blocked, this component is not amenable to targeted pharmacological inhibition.

The second component of edema is driven by osmotically active solutes, in this case ions and proteins, that are transported from the vascular compartment into the injured brain, to form ionic and vasogenic edema. Involvement of endothelial transporters/co-transporters such as SUR1-TRPM4 in this process suggests that this component may be subject to pharmacological inhibition. In a rat contusion-TBI model, with an increase in brain water from 78.5 to 80.8%, Zweckberger and colleagues^14^ reported that glibenclamide decreased excess water down to 80.5%, or by 15%. In a mouse contusion-TBI model complicated by hypotension, with an increase in brain water from 78.3 to 80.4%, Jha and colleagues^17^ reported that glibenclamide did not reduce ipsilateral excess water, but that contralateral excess water, which was increased from 78.3 to 78.6%, was eliminated by the drug. In a mouse contusion-TBI model, Xu and colleagues^15^ reported that glibenclamide reduced Evans blue extravasation by 35%. Glibenclamide was shown recently in a human clinical trial to reduce brain swelling from edema after ischemic stroke,^21^ but a comparable effect on edema in human TBI remains to be examined. In the present study, our experiments with AS-ODNs were designed explicitly to address HPC and its role in hemispheric swelling, not the subsequent development of edema. Future studies will examine the effects of AS-ODNs on edema formation in a rat contusion-TBI model.

## Conclusion

In humans and rats, contusion-TBI is accompanied by upregulation of SUR1-TRPM4 in endothelium, astrocytes and microglia/macrophages, and of K_ATP_ (SUR1-KIR6.2) in astrocytes and microglia/macrophages, with cell-type specific expression being different in the lesion core versus the penumbra. *In vivo* experiments with AS-ODNs show that SUR1-TRPM4 plays a critical role in HPC and in early brain swelling, whereas a role for K_ATP_ in TBI has yet to be identified.

## References

[1] Graham D.I., Adams J.H., Nicoll J.A., Maxwell W.L., and Gennarelli T.A. (1995). The nature, distribution and causes of traumatic brain injury. Brain Pathol. 5, 397–406 8974622 10.1111/j.1750-3639.1995.tb00618.x

[2] Saatman K.E., Duhaime A.C., Bullock R., Maas A.I., Valadka A., and Manley G.T.; Workshop Scientific Team and Advisory Panel Members. (2008). Classification of traumatic brain injury for targeted therapies. J. Neurotrauma 25, 719–738 18627252 10.1089/neu.2008.0586 PMC2721779

[3] Cepeda S., Gomez P.A., Castano-Leon A.M., Martinez-Perez R., Munarriz P.M., and Lagares A. (2015). Traumatic intracerebral hemorrhage: risk factors associated with progression. J. Neurotrauma 32, 1246–1253 25752340 10.1089/neu.2014.3808

[4] Simard J.M., Kilbourne M., Tsymbalyuk O., Tosun C., Caridi J., Ivanova S., Keledjian K., Bochicchio G., and Gerzanich V. (2009). Key role of sulfonylurea receptor 1 in progressive secondary hemorrhage after brain contusion. J. Neurotrauma 26, 2257–2267 19604096 10.1089/neu.2009.1021 PMC2824216

[5] Kurland D., Hong C., Aarabi B., Gerzanich V., and Simard J.M. (2012). Hemorrhagic progression of a contusion after traumatic brain injury: a review. J. Neurotrauma 29, 19–31 21988198 10.1089/neu.2011.2122 PMC3253310

[6] Maegele M., Schochl H., Menovsky T., Marechal H., Marklund N., Buki A., and Stanworth S. (2017). Coagulopathy and haemorrhagic progression in traumatic brain injury: advances in mechanisms, diagnosis, and management. Lancet Neurol. 16, 630–647 28721927 10.1016/S1474-4422(17)30197-7

[7] Dietrich W.D., Alonso O., and Halley M. (1994). Early microvascular and neuronal consequences of traumatic brain injury: a light and electron microscopic study in rats. J. Neurotrauma 11, 289–301 7996583 10.1089/neu.1994.11.289

[8] Alahmadi H., Vachhrajani S., and Cusimano M.D. (2010). The natural history of brain contusion: an analysis of radiological and clinical progression. J. Neurosurg. 112, 1139–1145 19575576 10.3171/2009.5.JNS081369

[9] Carnevale J.A., Segar D.J., Powers A.Y., Shah M., Doberstein C., Drapcho B., Morrison J.F., Williams J.R., Collins S., Monteiro K., and Asaad W.F. (2018). Blossoming contusions: identifying factors contributing to the expansion of traumatic intracerebral hemorrhage. J. Neurosurg. 1–12 10.3171/2017.7.JNS17988 29303442

[10] Nelson D.W., Nystrom H., MacCallum R.M., Thornquist B., Lilja A., Bellander B.M., Rudehill A., Wanecek M., and Weitzberg E. (2010). Extended analysis of early computed tomography scans of traumatic brain injured patients and relations to outcome. J. Neurotrauma 27, 51–64 19698072 10.1089/neu.2009.0986

[11] Jacobs B., Beems T., van der Vliet T.M., Diaz-Arrastia R.R., Borm G.F., and Vos P.E. (2011). Computed tomography and outcome in moderate and severe traumatic brain injury: hematoma volume and midline shift revisited. J. Neurotrauma 28, 203–215 21294647 10.1089/neu.2010.1558

[12] Patel A.D., Gerzanich V., Geng Z., and Simard J.M. (2010). Glibenclamide reduces hippocampal injury and preserves rapid spatial learning in a model of traumatic brain injury. J. Neuropathol. Exp. Neurol. 69, 1177–1190 21107131 10.1097/NEN.0b013e3181fbf6d6

[13] Martinez-Valverde T., Vidal-Jorge M., Martinez-Saez E., Castro L., Arikan F., Cordero E., Radoi A., Poca M.A., Simard J.M., and Sahuquillo J. (2015). Sulfonylurea Receptor 1 in humans with post-traumatic brain contusions. J. Neurotrauma 32, 1478–1487 26398596 10.1089/neu.2014.3706 PMC4589328

[14] Zweckberger K., Hackenberg K., Jung C.S., Hertle D.N., Kiening K.L., Unterberg A.W., and Sakowitz O.W. (2014). Glibenclamide reduces secondary brain damage after experimental traumatic brain injury. Neuroscience 272, 199–206 24792709 10.1016/j.neuroscience.2014.04.040

[15] Xu Z.M., Yuan F., Liu Y.L., Ding J., and Tian H.L. (2017). Glibenclamide attenuates blood-brain barrier disruption in adult mice after traumatic brain injury. J. Neurotrauma 34, 925–933 27297934 10.1089/neu.2016.4491

[16] Kochanek P.M., Bramlett H.M., Dixon C.E., Dietrich W.D., Mondello S., Wang K.K.W., Hayes R.L., Lafrenaye A., Povlishock J.T., Tortella F.C., Poloyac S.M., Empey P., and Shear D.A. (2018). Operation brain trauma therapy: 2016 update. Mil. Med. 183, 303–312 29635589 10.1093/milmed/usx184

[17] Jha R., Molyneaux B.J., Jackson T.C., Wallisch J., Park S.Y., Poloyac S.M., Vagni V.A., Janesko-Feldman K.L., Hoshitsuki K., Minnigh M.B., and Kochanek P.M. (2018). Glibenclamide produces region-dependent effects on cerebral edema in a combined injury model of traumatic brain injury and hemorrhagic shock in mice. J. Neurotrauma 35, 2125–2135 29648981 10.1089/neu.2016.4696 PMC6098411

[18] Jha R.M., Puccio A.M., Chou S.H., Chang C.H., Wallisch J.S., Molyneaux B.J., Zusman B.E., Shutter L.A., Poloyac S.M., Janesko-Feldman K.L., Okonkwo D.O., and Kochanek P.M. (2017). Sulfonylurea receptor-1: a novel biomarker for cerebral edema in severe traumatic brain injury. Crit. Care Med. 45, e255–e264 27845954 10.1097/CCM.0000000000002079 PMC5550829

[19] Jha R.M., Puccio A.M., Okonkwo D.O., Zusman B.E., Park S.Y., Wallisch J., Empey P.E., Shutter L.A., Clark R.S., Kochanek P.M., and Conley Y.P. (2017). ABCC8 single nucleotide polymorphisms are associated with cerebral edema in severe TBI. Neurocrit. Care 26, 213–224 27677908 10.1007/s12028-016-0309-z PMC5550833

[20] Jha R.M., Koleck T.A., Puccio A.M., Okonkwo D.O., Park S.Y., Zusman B.E., Clark R.S.B., Shutter L.A., Wallisch J.S., Empey P.E., Kochanek P.M., and Conley Y.P. (2018). Regionally clustered ABCC8 polymorphisms in a prospective cohort predict cerebral oedema and outcome in severe traumatic brain injury. J. Neurol. Neurosurg. Psychiatry pii, 10.1136/jnnp-2017-317741 PMC6181785 29674479

[21] Sheth K.N., Elm J.J., Molyneaux B.J., Hinson H., Beslow L.A., Sze G.K., Ostwaldt A.C., Del Zoppo G.J., Simard J.M., Jacobson S., and Kimberly W.T. (2016). Safety and efficacy of intravenous glyburide on brain swelling after large hemispheric infarction (GAMES-RP): a randomised, double-blind, placebo-controlled phase 2 trial. Lancet Neurol. 15, 1160–1169 27567243 10.1016/S1474-4422(16)30196-X

[22] Khalili H., Derakhshan N., Niakan A., Ghaffarpasand F., Salehi M., Eshraghian H., Shakibafard A., and Zahabi B. (2017). Effects of oral glibenclamide on brain contusion volume and functional outcome of patients with moderate and severe traumatic brain injuries: a randomized double-blind placebo-controlled clinical trial. World Neurosurg. 101, 130–136 28185976 10.1016/j.wneu.2017.01.103

[23] Noma A. (1983). ATP-regulated K+ channels in cardiac muscle. Nature 305, 147–148 6310409 10.1038/305147a0

[24] Bryan J., Munoz A., Zhang X., Dufer M., Drews G., Krippeit-Drews P., and Aguilar-Bryan L. (2007). ABCC8 and ABCC9: ABC transporters that regulate K+ channels. Pflugers Arch. 453, 703–718 16897043 10.1007/s00424-006-0116-z

[25] Aittoniemi J., Fotinou C., Craig T.J., de Wet H., Proks P., and Ashcroft F.M. (2009). Review. SUR1: a unique ATP-binding cassette protein that functions as an ion channel regulator. Philos. Trans. R. Soc. Lond. B Biol. Sci. 364, 257–267 18990670 10.1098/rstb.2008.0142 PMC2674095

[26] Woo S.K., Kwon M.S., Ivanov A., Gerzanich V., and Simard J.M. (2013). The sulfonylurea receptor 1 (Sur1)-transient receptor potential melastatin 4 (Trpm4) channel. J. Biol. Chem. 288, 3655–3667 23255597 10.1074/jbc.M112.428219 PMC3561583

[27] Gorse K., Lantzy M.K., Lee E.D., and Lafrenaye A.D. (2018). Trpm4 induces astrocyte swelling but not death after diffuse traumatic brain injury. J. Neurotrauma 15 Jul 2018; Epub ahead of print 10.1089/neu.2017.5275 PMC6016101 29390943

[28] Castro L., Noelia M., Vidal-Jorge M., Sanchez-Ortiz D., Gandara D., Martinez-Saez E., Cicuendez M., Poca M.A.D., Simard J.M., and Sahuquillo J. (2018). Kir6.2, the pore-forming subunit of ATP-sensitive K+ channels, is overexpressed in human post-traumatic brain contusions. J. Neurotrauma 2018 Jul 24; Epub ahead of print 10.1089/neu.2017.5619 PMC7872003 29737232

[29] Aarabi B., Hesdorffer D.C., Simard J.M., Ahn E.S., Aresco C., Eisenberg H.M., McCunn M., and Scalea T. (2009). Comparative study of decompressive craniectomy after mass lesion evacuation in severe head injury. Neurosurgery 64, 927–39 19287327 10.1227/01.NEU.0000341907.30831.D2

[30] Mehta R.I., Ivanova S., Tosun C., Castellani R.J., Gerzanich V., and Simard J.M. (2013). Sulfonylurea receptor 1 expression in human cerebral infarcts. J. Neuropathol. Exp. Neurol. 72, 871–883 23965746 10.1097/NEN.0b013e3182a32e40 PMC3771575

[31] Uhlen M., Bandrowski A., Carr S., Edwards A., Ellenberg J., Lundberg E., Rimm D.L., Rodriguez H., Hiltke T., Snyder M., and Yamamoto T. (2016). A proposal for validation of antibodies. Nat. Methods 13, 823–827 27595404 10.1038/nmeth.3995 PMC10335836

[32] Yan F.F., Lin C.W., Cartier E.A., and Shyng S.L. (2005). Role of ubiquitin-proteasome degradation pathway in biogenesis efficiency of {beta}-cell ATP-sensitive potassium channels. Am. J. Physiol. Cell Physiol. 289, C1351–C1359 15987767 10.1152/ajpcell.00240.2005 PMC1350484

[33] Konig P., Krasteva G., Tag C., Konig I.R., Arens C., and Kummer W. (2006). FRET-CLSM and double-labeling indirect immunofluorescence to detect close association of proteins in tissue sections. Lab. Invest. 86, 853–864 16783395 10.1038/labinvest.3700443

[34] Tosun C., Kurland D.B., Mehta R., Castellani R.J., deJong J.L., Kwon M.S., Woo S.K., Gerzanich V., and Simard J.M. (2013). Inhibition of the Sur1-Trpm4 channel reduces neuroinflammation and cognitive impairment in subarachnoid hemorrhage. Stroke 44, 3522–3528 24114458 10.1161/STROKEAHA.113.002904 PMC3894855

[35] Mehta R.I., Tosun C., Ivanova S., Tsymbalyuk N., Famakin B.M., Kwon M.S., Castellani R.J., Gerzanich V., and Simard J.M. (2015). Sur1-Trpm4 Cation Channel Expression in Human Cerebral Infarcts. J. Neuropathol. Exp. Neurol. 74, 835–849 26172285 10.1097/NEN.0000000000000223 PMC4620032

[36] Simard J.M., Woo S.K., Tsymbalyuk N., Voloshyn O., Yurovsky V., Ivanova S., Lee R., and Gerzanich V. (2012). Glibenclamide-10-h treatment window in a clinically relevant model of stroke. Transl. Stroke Res. 3, 286–295 22707989 10.1007/s12975-012-0149-x PMC3362710

[37] Simard J.M., Chen M., Tarasov K.V., Bhatta S., Ivanova S., Melnitchenko L., Tsymbalyuk N., West G.A., and Gerzanich V. (2006). Newly expressed SUR1-regulated NC(Ca-ATP) channel mediates cerebral edema after ischemic stroke. Nat. Med. 12, 433–440 16550187 10.1038/nm1390 PMC2740734

[38] Simard J.M., Tsymbalyuk O., Ivanov A., Ivanova S., Bhatta S., Geng Z., Woo S.K., and Gerzanich V. (2007). Endothelial sulfonylurea receptor 1-regulated NC Ca-ATP channels mediate progressive hemorrhagic necrosis following spinal cord injury. J. Clin. Invest. 117, 2105–2113 17657312 10.1172/JCI32041 PMC1924498

[39] Gerzanich V., Woo S.K., Vennekens R., Tsymbalyuk O., Ivanova S., Ivanov A., Geng Z., Chen Z., Nilius B., Flockerzi V., Freichel M., and Simard J.M. (2009). De novo expression of Trpm4 initiates secondary hemorrhage in spinal cord injury. Nat. Med. 15, 185–191 19169264 10.1038/nm.1899 PMC2730968

[40] Lamensdorf I., Meiri N., Harvey-White J., Jacobowitz D.M., and Kopin I.J. (1999). Kir6.2 oligoantisense administered into the globus pallidus reduces apomorphine-induced turning in 6-OHDA hemiparkinsonian rats. Brain Res. 818, 275–284 10082813 10.1016/s0006-8993(98)01290-6

[41] Galderisi U., Cascino A., and Giordano A. (1999). Antisense oligonucleotides as therapeutic agents. J. Cell. Physiol. 181, 251–257 10497304 10.1002/(SICI)1097-4652(199911)181:2<251::AID-JCP7>3.0.CO;2-D

[42] Simard J.M., Yurovsky V., Tsymbalyuk N., Melnichenko L., Ivanova S and Gerzanich V. (2009). Protective effect of delayed treatment with low-dose glibenclamide in three models of ischemic stroke. Stroke 40, 604–609 19023097 10.1161/STROKEAHA.108.522409 PMC2744391

[43] Liu D., Smith C.L., Barone F.C., Ellison J.A., Lysko P.G., Li K., and Simpson I.A. (1999). Astrocytic demise precedes delayed neuronal death in focal ischemic rat brain. Brain research. Mol. Brain Res. 68, 29–41 10320781 10.1016/s0169-328x(99)00063-7

[44] Zhao X., Ahram A., Berman R.F., Muizelaar J.P., and Lyeth B.G. (2003). Early loss of astrocytes after experimental traumatic brain injury. Glia 44, 140–152 14515330 10.1002/glia.10283

[45] Abu Hamdeh S., Shevchenko G., Mi J., Musunuri S., Bergquist J., and Marklund N. (2018). Proteomic differences between focal and diffuse traumatic brain injury in human brain tissue. Sci. Rep. 8, 6807 29717219 10.1038/s41598-018-25060-0 PMC5931620

[46] Vinogradov S.V., Batrakova E.V., and Kabanov A.V. (2004). Nanogels for oligonucleotide delivery to the brain. Bioconjug. Chem. 15, 50–60 14733583 10.1021/bc034164r PMC2837941

[47] Baskaya M.K., Rao A.M., Dogan A., Donaldson D., and Dempsey R.J. (1997). The biphasic opening of the blood-brain barrier in the cortex and hippocampus after traumatic brain injury in rats. Neurosci. Lett. 226, 33–36 9153635 10.1016/s0304-3940(97)00239-5

[48] Simard J.M., Woo S.K., Norenberg M.D., Tosun C., Chen Z., Ivanova S., Tsymbalyuk O., Bryan J., Landsman D., and Gerzanich V. (2010). Brief suppression of Abcc8 prevents autodestruction of spinal cord after trauma. Sci. Transl. Med. 2, 28ra29 10.1126/scitranslmed.3000522 PMC2903041 20410530

[49] Soundarapandian M.M., Zhong X., Peng L., Wu D., and Lu Y. (2007). Role of K(ATP) channels in protection against neuronal excitatory insults. J. Neurochem. 103, 1721–1729 17944875 10.1111/j.1471-4159.2007.04963.x

[50] Sun H.S. and Feng Z.P. (2013). Neuroprotective role of ATP-sensitive potassium channels in cerebral ischemia. Acta Pharmacol. Sin. 34, 24–32 23123646 10.1038/aps.2012.138 PMC4086509

[51] Szeto V., Chen N.H., Sun H.S., and Feng Z.P. (2018). The role of KATP channels in cerebral ischemic stroke and diabetes. Acta Pharmacol. Sin. 39, 683–694 29671418 10.1038/aps.2018.10 PMC5943906

[52] Yamada K., Ji J.J., Yuan H., Miki T., Sato S., Horimoto N., Shimizu T., Seino S., and Inagaki N. (2001). Protective role of ATP-sensitive potassium channels in hypoxia-induced generalized seizure. Science 292, 1543–1546 11375491 10.1126/science.1059829

[53] Sun H.S., Feng Z.P., Miki T., Seino S and French R.J. (2006). Enhanced neuronal damage after ischemic insults in mice lacking Kir6.2-containing ATP-sensitive K+ channels. J. Neurophysiol 95, 2590–2601 16354731 10.1152/jn.00970.2005

[54] Heron-Milhavet L., Xue-Jun Y., Vannucci S.J., Wood T.L., Willing L.B., Stannard B., Hernandez-Sanchez C., Mobbs C., Virsolvy A., and LeRoith D. (2004). Protection against hypoxic-ischemic injury in transgenic mice overexpressing Kir6.2 channel pore in forebrain. Mol. Cell. Neurosci. 25, 585–593 15080888 10.1016/j.mcn.2003.10.012

[55] Dunn-Meynell A.A., Rawson N.E., and Levin B.E. (1998). Distribution and phenotype of neurons containing the ATP-sensitive K+ channel in rat brain. Brain Res. 814, 41–54 9838037 10.1016/s0006-8993(98)00956-1

[56] Zhou M., Tanaka O., Suzuki M., Sekiguchi M., Takata K., Kawahara K., and Abe H. (2002). Localization of pore-forming subunit of the ATP-sensitive K(+)-channel, Kir6.2, in rat brain neurons and glial cells. Brain research. Mol. Brain Res. 101, 23–32 12007828 10.1016/s0169-328x(02)00137-7

[57] Zhou M., Tanaka O., Sekiguchi M., Sakabe K., Anzai M., Izumida I., Inoue T., Kawahara K., and Abe H. (1999). Localization of the ATP-sensitive potassium channel subunit (Kir6. 1/uK(ATP)-1) in rat brain. Brain Res, Mol, Brain Res. 74, 15–25 10640672 10.1016/s0169-328x(99)00232-6

[58] Thomzig A., Wenzel M., Karschin C., Eaton M.J., Skatchkov S.N., Karschin A., and Veh R.W. (2001). Kir6.1 is the principal pore-forming subunit of astrocyte but not neuronal plasma membrane K-ATP channels. Mol. Cell. Neurosci. 18, 671–690 11749042 10.1006/mcne.2001.1048

[59] Griffith C.M., Xie M.X., Qiu W.Y., Sharp A.A., Ma C., Pan A., Yan X.X., and Patrylo P.R. (2016). Aberrant expression of the pore-forming KATP channel subunit Kir6.2 in hippocampal reactive astrocytes in the 3xTg-AD mouse model and human Alzheimer's disease. Neuroscience 336, 81–101 27586053 10.1016/j.neuroscience.2016.08.034

[60] Ningaraj N.S., Rao M.K., and Black K.L. (2003). Adenosine 5'-triphosphate-sensitive potassium channel-mediated blood-brain tumor barrier permeability increase in a rat brain tumor model. Cancer Res. 63, 8899–8911 14695207

[61] Ploug K.B., Edvinsson L., Olesen J., and Jansen-Olesen I. (2006). Pharmacological and molecular comparison of K(ATP) channels in rat basilar and middle cerebral arteries. Eur. J. Pharmacol. 553, 254–262 17101127 10.1016/j.ejphar.2006.09.053

[62] Santa N., Kitazono T., Ago T., Ooboshi H., Kamouchi M., Wakisaka M., Ibayashi S., and Iida M. (2003). ATP-sensitive potassium channels mediate dilatation of basilar artery in response to intracellular acidification in vivo. Stroke 34, 1276–1280 12677015 10.1161/01.STR.0000068171.01248.97

[63] Jansen-Olesen I., Mortensen C.H., El-Bariaki N., and Ploug K.B. (2005). Characterization of K(ATP)-channels in rat basilar and middle cerebral arteries: studies of vasomotor responses and mRNA expression. Eur. J. Pharmacol. 523, 109–118 16226739 10.1016/j.ejphar.2005.08.028

[64] Fujita A. and Kurachi Y. (2000). Molecular aspects of ATP-sensitive K+ channels in the cardiovascular system and K+ channel openers. Pharmacol. therapeutics 85, 39–53 10.1016/s0163-7258(99)00050-9 10674713

[65] Simard J.M., Woo S.K., Aarabi B., and Gerzanich V. (2013). The Sur1-Trpm4 channel in spinal cord injury. J. Spine Suppl 4 10.4172/2165-7939.S4-002 PMC4019017 24834370

[66] Makar T.K., Gerzanich V., Nimmagadda V.K., Jain R., Lam K., Mubariz F., Trisler D., Ivanova S., Woo S.K., Kwon M.S., Bryan J., Bever C.T., and Simard J.M. (2015). Silencing of Abcc8 or inhibition of newly upregulated Sur1-Trpm4 reduce inflammation and disease progression in experimental autoimmune encephalomyelitis. J. Neuroinflammation 12, 210 26581714 10.1186/s12974-015-0432-3 PMC4652344

[67] Kurland D.B., Gerzanich V., Karimy J.K., Woo S.K., Vennekens R., Freichel M., Nilius B., Bryan J., and Simard J.M. (2016). The Sur1-Trpm4 channel regulates NOS2 transcription in TLR4-activated microglia. J. Neuroinflammation 13, 130 27246103 10.1186/s12974-016-0599-2 PMC4888589

[68] Stokum J.A., Kwon M.S., Woo S.K., Tsymbalyuk O., Vennekens R., Gerzanich V., and Simard J.M. (2018). SUR1-TRPM4 and AQP4 form a heteromultimeric complex that amplifies ion/water osmotic coupling and drives astrocyte swelling. Glia 66, 108–125 28906027 10.1002/glia.23231 PMC5759053

[69] Ammala C., Bokvist K., Galt S., and Rorsman P. (1991). Inhibition of ATP-regulated K(+)-channels by a photoactivatable ATP-analogue in mouse pancreatic beta-cells. Biochim. Biophys. Acta 1092, 347–349 2049403 10.1016/s0167-4889(97)90011-2

[70] Bokvist K., Ammala C., Ashcroft F.M., Berggren P.O., Larsson O., and Rorsman P. (1991). Separate processes mediate nucleotide-induced inhibition and stimulation of the ATP-regulated K(+)-channels in mouse pancreatic beta-cells. Proc. Biol. Sci. 243, 139–144 1676517 10.1098/rspb.1991.0022

[71] Chen M. and Simard J.M. (2001). Cell swelling and a nonselective cation channel regulated by internal Ca2+ and ATP in native reactive astrocytes from adult rat brain. J. Neurosci. 21, 6512–6521 11517240 10.1523/JNEUROSCI.21-17-06512.2001 PMC6763097

[72] Cheng H., Beck A., Launay P., Gross S.A., Stokes A.J., Kinet J.P., Fleig A., and Penner R. (2007). TRPM4 controls insulin secretion in pancreatic beta-cells. Cell Calcium 41, 51–61 16806463 10.1016/j.ceca.2006.04.032 PMC5663640

[73] Hashiguchi H., Sheng Z., Routh V., Gerzanich V., Simard J.M., and Bryan J. (2017). Direct versus indirect actions of ghrelin on hypothalamic NPY neurons. PloS one 12, e0184261 28877214 10.1371/journal.pone.0184261 PMC5587286

[74] Marigo V., Courville K., Hsu W.H., Feng J.M., and Cheng H. (2009). TRPM4 impacts on Ca2+ signals during agonist-induced insulin secretion in pancreatic beta-cells. Mol. Cell. Endocrinol. 299, 194–203 19063936 10.1016/j.mce.2008.11.011

[75] Jayakumar A.R., Tong X.Y., Ruiz-Cordero R., Bregy A., Bethea J.R., Bramlett H.M., and Norenberg M.D. (2014). Activation of NF-kappaB mediates astrocyte swelling and brain edema in traumatic brain injury. J. Neurotrauma 31, 1249–1257 24471369 10.1089/neu.2013.3169 PMC4108982

[76] Marmarou A., Maset A.L., Ward J.D., Choi S., Brooks D., Lutz H.A., Moulton R.J., Muizelaar J.P., DeSalles A., and Young H.F. (1987). Contribution of CSF and vascular factors to elevation of ICP in severely head-injured patients. J. Neurosurg. 66, 883–890 3572518 10.3171/jns.1987.66.6.0883

[77] Kelly D.F., Kordestani R.K., Martin N.A., Nguyen T., Hovda D.A., Bergsneider M., McArthur D.L., and Becker D.P. (1996). Hyperemia following traumatic brain injury: relationship to intracranial hypertension and outcome. J. Neurosurg. 85, 762–771 8893712 10.3171/jns.1996.85.5.0762

[78] Katayama Y. and Kawamata T. (2003). Edema fluid accumulation within necrotic brain tissue as a cause of the mass effect of cerebral contusion in head trauma patients. Acta Neurochir. Suppl. 86, 323–327 14753461 10.1007/978-3-7091-0651-8_69

[79] Kawamata T., Mori T., Sato S., and Katayama Y. (2007). Tissue hyperosmolality and brain edema in cerebral contusion. Neurosurg. Focus 22, E5 10.3171/foc.2007.22.5.6 17613236

